# The TFEB-TGIF1 axis regulates EMT in mouse epicardial cells

**DOI:** 10.1038/s41467-022-32855-3

**Published:** 2022-09-03

**Authors:** Elena Astanina, Gabriella Doronzo, Davide Corà, Francesco Neri, Salvatore Oliviero, Tullio Genova, Federico Mussano, Emanuele Middonti, Edoardo Vallariello, Chiara Cencioni, Donatella Valdembri, Guido Serini, Federica Limana, Eleonora Foglio, Andrea Ballabio, Federico Bussolino

**Affiliations:** 1 Department of Oncology, University of Torino, Torino, Italy; 2 Candiolo Cancer Institute-IRCCS-FPO, Candiolo, Italy; 3 Department of Translational Medicine, Università degli Studi del Piemonte Orientale, Novara, Italy; 4 Department of Life Sciences and Systems Biology, University of Torino, Torino, Italy; 5 CIR Dental School, Department of Surgical Sciences, University of Torino, Via Nizza 230, 10126 Turin, Italy; 6 Institute for Systems Analysis and Computer Science “A. Ruberti”, National Research Council (IASI-CNR), Rome, Italy; 7 San Raffaele Open University, Rome, Italy; 8 Laboratory of Cellular and Molecular Pathology, IRCCS San Raffaele Pisana, Rome, Italy; 9 Technoscience, Parco Scientifico e Tecnologico Pontino, 04100 Latina, Italy; 10 Telethon Institute of Genetics and Medicine (TIGEM), Pozzuoli, Italy; 11 Medical Genetics Unit, Department of Medical and Translational Science, Federico II University, Naples, Italy; 12 Department of Molecular and Human Genetics, Baylor College of Medicine, Houston, TX USA; 13 Jan and Dan Duncan Neurological Research Institute, Texas Children’s Hospital, Houston, TX USA

**Keywords:** Epithelial-mesenchymal transition, Cell lineage, Differentiation, Organogenesis, Transcription

## Abstract

Epithelial-mesenchymal transition (EMT) is a complex and pivotal process involved in organogenesis and is related to several pathological processes, including cancer and fibrosis. During heart development, EMT mediates the conversion of epicardial cells into vascular smooth muscle cells and cardiac interstitial fibroblasts. Here, we show that the oncogenic transcription factor EB (TFEB) is a key regulator of EMT in epicardial cells and that its genetic overexpression in mouse epicardium is lethal due to heart defects linked to impaired EMT. TFEB specifically orchestrates the EMT-promoting function of transforming growth factor (TGF) β, and this effect results from activated transcription of thymine-guanine-interacting factor (TGIF)1, a TGFβ/Smad pathway repressor. The *Tgif1* promoter is activated by TFEB, and in vitro and in vivo findings demonstrate its increased expression when *Tfeb* is overexpressed. Furthermore, *Tfeb* overexpression in vitro prevents TGFβ-induced EMT, and this effect is abolished by *Tgif1* silencing. *Tfeb* loss of function, similar to that of *Tgif1*, sensitizes cells to TGFβ, inducing an EMT response to low doses of TGFβ. Together, our findings reveal an unexpected function of TFEB in regulating EMT, which might provide insights into injured heart repair and control of cancer progression.

## Abstract (web summary)

Epithelial-mesenchymal transition (EMT) is a complex process involved in organogenesis. Here, the authors show that the transcription factor EB (TFEB) regulates EMT in epicardium during heart development by tuning sensitivity to TGFβ signaling.

## Introduction

Epithelial-mesenchymal transition (EMT) is a fundamental biological program based on cell plasticity that characterizes embryonic development and maintenance of tissue homeostasis in adult life. In physiological settings, EMT promotes the transition from static and polarized epithelial cells to a motogenic and mesenchymal phenotype, which allows population of different regions of the embryo and promotion of organogenesis and tissue renewal. When aberrantly activated, EMT enhances the pathogenesis of many chronic degenerative diseases, including cancer and fibrosis^1^.

A paradigmatic example of physiological EMT is the contribution of the epicardium to myocardial organogenesis. The epicardium is a mesothelial layer covering the heart that originates from the proepicardium (PE), a transient and heterogenous mesodermal structure localized at the venous pole of the heart. Proepicardial cells attach, grow and cover the naked myocardium to form an outer epithelial layer^2^. Subsequently, some of the epicardial cells undergo EMT and give rise to the so‐called epicardium‐derived cells (EPDCs), which migrate into the myocardium and differentiate into fibroblasts and the vascular smooth muscle cells (vSMCs) of coronaries^3–5^. In adults, myocardial injury may reactivate epicardial EMT, contributing to tissue repair^6,7^.

The complexity of epicardial EMT as well as the invasion of EPDCs into the myocardium and their lineage commitment is orchestrated by external cues and specific transcriptional patterns. Many signaling pathways acting in an autocrine and paracrine manner have been shown to cooperate with different regulators of epicardial development, including retinoic acid^8^, fibroblast growth factor^9^, transforming-growth factor (TGF) β^10^, and platelet-derived growth factor (PDGF)^11,12^. The TGFβ pathway is required for the induction of epicardial EMT and further differentiation of EPDCs into vSMCs^10,13,14^. TGFβ signals through a heterodimeric complex between Type I and Type II serine/threonine kinase receptors via phosphorylation and therefore activation of receptor-regulated SMAD (R-SMAD) proteins, such as SMAD2 and SMAD3. Phosphorylated R-SMADs form complexes with SMAD4 and translocate to the nucleus to initiate specific transcriptional programs regulated by the recruitment of coactivators and corepressors^15^.

Epicardial EMT and the differentiation of the EPDC lineage also require the organization of dynamic circuits between a discrete number of transcription factors (TFs) triggered by cell-autonomous and cell-nonautonomous mechanisms^16^. Some of these TFs, such as Twist and Snails, regulate EMT in all tissues^1,17^, while others are restricted to the epicardium. The use of genetic Cre-loxP-based mouse models has allowed to identify the role of the Wilms Tumor-1 (WT1)^18,19^, Tcf21^20^, YAP^21^, serum response factor^22^, Nfatc1^23^, Notch-Rbpj^24^, and Tbx18^25^ TFs in epicardial EMT.

The oncogenic TF EB (TFEB) belongs to the microphthalmia gene family of bHLH-leucine zipper TFs, which includes microphthalmia-associated TF (MITF), TF E3 (TFE3), and TF EC (TFEC). TFEB resides in the cytosol and translocates to the nucleus to regulate transcription. Its nucleocytoplasmic shuttling is regulated by post‐translational modifications, including phosphorylation/dephosphorylation by mTOR on S122, S142, and S211^26,27^. TFEB was originally shown to be translocated in a subset of renal carcinomas^28^ and then demonstrated to be deregulated in several cancers^29^. The current findings clearly support the existence of an important pathway by which TFEB acts as a master regulator of lysosomal and autophagosome biogenesis and represents a molecular tool to adapt cells to stress, including starvation and energy depletion^30–32^. More recent results have demonstrated wider regulatory activities of TFEB, which most likely emerge in specific cellular contexts. Independent of the autophagic pathway, TFEB has been reported to regulate the cell cycle^33,34^, metabolism^35–37^, and vascular^33,38^ and immune functions^39^. Cell lineage commitment and differentiation^34,40–43^ and embryonic development^33,38^ are emerging areas to contextualize the role of both autophagic and noncanonical pathways orchestrated by TFEB.

Here, we demonstrate a role for TFEB in the regulation of epicardial EMT. Using a *Tfeb-EGFP*-expressing mouse model, we show that *Tfeb* is expressed in epicardial cells and downregulated during EMT, specifically upon activation of TGFβ1 signaling. *Tfeb* overexpression, both in mouse epicardium and in cellular models, inhibits EMT, while *Tfeb* loss of function sensitizes cells to TGFβ. Mechanistically, TFEB directly promotes the transcription of thymine-guanine-interacting factor (TGIF)1, a homeodomain protein of the TALE subfamily, which is recruited to activated SMAD2/SMAD3 complexes and represses the TGFβ-mediated transcriptional machinery by multiple mechanisms^44,45^.

## Results

### Dynamic *Tfeb* expression in embryonic mouse epicardial cells

**Fig. 1:**
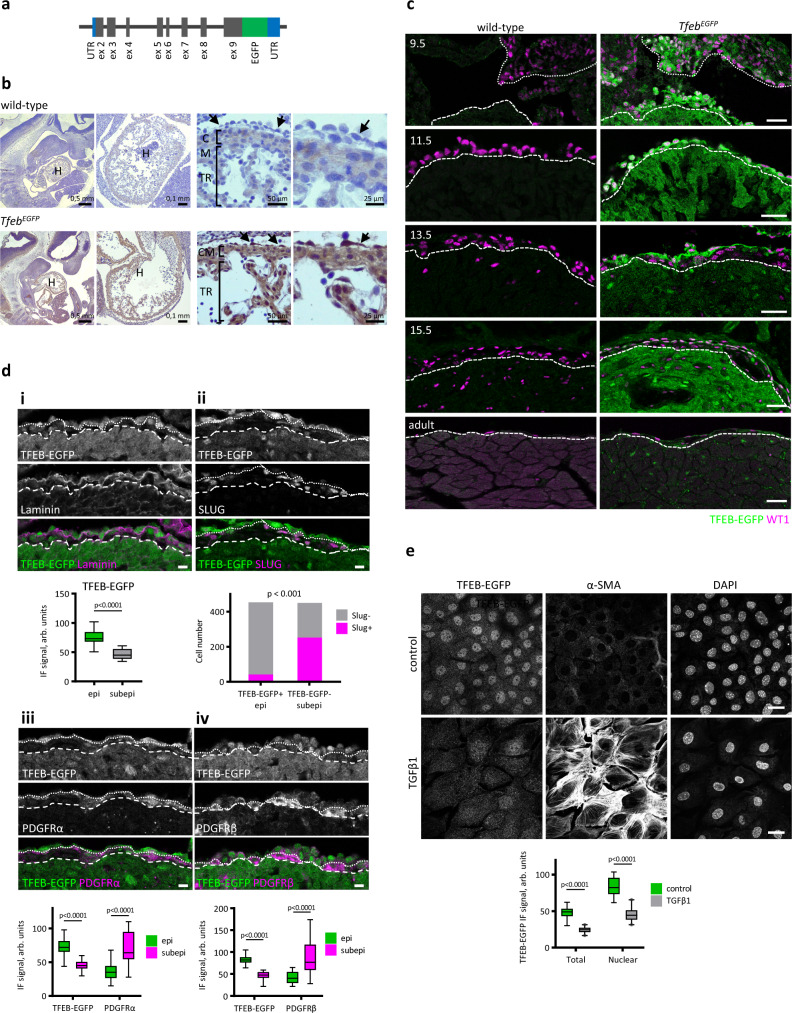
*Tfeb* is expressed in murine epicardium and is downregulated in epicardial cells undergoing EMT. **a** Scheme of the constitutive knock-in *Tfeb-EGFP* allele (C57BL/6NTac-Tfeb^*tm3205(EGFP)Art*^ mouse). **b** EGFP immunohistochemical analysis of *Tfeb*^*EGFP*^ and wild-type mouse embryos at E11.5. Four embryos for genotype were analyzed. H heart, CM compact myocardium, TR trabeculae, arrows indicate the epicardial layer. Scale bars length is indicated in the figure. **c** Immunostaining for EGFP (green) and WT1 (magenta) in the hearts of wild-type and *Tfeb*^*EGFP*^ mouse embryos at the indicated embryonic day and in adult mice. The overlay of EGFP and cardiac troponin T (cTnT) immunostainings is shown in Supplementary Fig. 2a. The dotted line surrounds the proepicardium at E9.5. The dashed line indicates the myocardium surface. Four embryos for genotype were analyzed. The scale bar is 25 μm. **d** Immunostaining for EGFP (green) and laminin, PDGFRα, PDGFRβ, and SLUG (magenta) in the hearts of E13.5 *Tfeb*^*EGFP*^ embryos. The overlays of EGFP, laminin, PDGFRα, PDGFRβ, SLUG immunstainings with nuclei (DAPI) staining are shown in Supplementary Fig. 2b. The dashed line indicates the myocardium surface, and the dotted line indicates the epicardium-subepicardium border. The scale bar is 25 μm. Image quantification was performed as follows. **i** Cells above the laminin-marked basement membrane were considered epicardial (epi), and cells between the basement membrane and myocardial surface were considered subepicardial (subepi). The average TFEB-EGFP immunofluorescence signal was measured in both cell populations. 4 embryos, with at least 4 images for an embryo, were used for the analysis. Box plots show the quartiles, the 5th and 95th percentiles (whiskers). Student’s paired two-tailed test *p* < 0.001. ii Cells positive for TFEB-EGFP were considered TFEB-EGFP + epicardial, cells negative for TFEB-EGFP and localized above myocardial surface–TFEB-EGFP-subepicardial. Nuclei positive and negative for SLUG were counted in both cell populations. Four embryos were used for the analysis. Values are shown as the number of nuclei, and Fisher’s exact test *p* value is indicated. iii, iv Cells positive for TFEB-EGFP were considered TFEB-EGFP + epicardial, cells negative for TFEB-EGFP and localized above myocardial surface–TFEB-EGFP- subepicardial. The average immunofluorescence signals of TFEB-EGFP, PDGFRα (iii), and PDGFRβ (iv) were measured in both populations. Four embryos, with at least four images for an embryo, were used for the analysis. Box plots show the quartiles, the 5th and 95th percentiles (whiskers). Student’s paired two-tailed test *p* < 0.001. **e** Top, immunofluorescence images of epicardial explants from E11.5 *Tfeb*^*EGFP*^ hearts treated with TGFβ1 for 48 h and immunostained for EGFP, α-SMA, and nuclei (DAPI). The scale bar is 25 μm. Bottom, quantification of the mean EGFP immunofluorescence signal in *Tfeb*^*EGFP*^ explants. Four explants for the genotype and 3–6 images for an explant were used for the analysis. Box plot shows the quartiles, the 5th and 95th percentiles (whiskers). Student’s two-tailed test *p* < 0.0001. Source data are provided as a Source Data file.

To explore *Tfeb* expression during embryonic development, we used a *Tfeb*^*EGFP*^ mouse model, in which the EGFP gene is knocked into the *Tfeb* locus, resulting in the fusion protein TFEB-EGFP (Fig. 1a)^33^. At E11.5, *Tfeb-EGFP* was expressed in the heart myocardium and at a particularly strong level in epicardial cells (Fig. 1b) and in placental trophoblasts and endothelial capillaries (Supplementary Fig. 1), as previously reported^33,38^. To further investigate whether *Tfeb* expression correlated with a particular stage of epicardial development, we analyzed EGFP expression in the heart tissues of wild-type and *Tfeb*^*EGFP*^ mice from E9.5 to E15.5 and in adults (Fig. 1c). *Tfeb* was specifically expressed in the nucleus and cytoplasm of WT1^+^ cells in the PE at E9.5 and in the epicardium up to E15.5. The expression decreased during development and was barely detectable in adult epicardial cells (Fig. 1c). To determine whether *Tfeb* expression differs in epicardial and epicardially derived cells undergoing EMT, we costained hearts at E13.5 for EGFP and markers of EMT (Fig. 1d). Epicardial cells localized above the basement membrane that was stained with an anti-laminin antibody, contained a significantly higher amount of TFEB-EGFP than EPDCs localized in the subepicardial space between the basement membrane and the myocardial surface (Fig. 1d i). Staining for the EMT-associated transcription factor Slug^46^ demonstrated that epicardial TFEB-EGFP^+^ cells had only 9% Slug^+^ nuclei, while subepicardial TFEB-EGFP^-^ cells had 56% Slug^+^ nuclei (Fig. 1d ii). Accordingly, subepicardial TFEB-EGFP^-^ cells were positive for PDGFRα or PDGFRβ, which are markers of fibroblast and SMC differentiation, respectively^11,12^ (Fig. 1d iii, iv). To further verify the correlation between *Tfeb* expression and the epithelial state of epicardial cells, we cultured epicardial explants from E11.5 *Tfeb*^*EGFP*^ hearts and induced EMT by TGFβ1 treatment. Figure 1e shows that TGFβ1 challenge resulted in a mesenchymal phenotype, as shown by the development of α-smooth muscle actin (SMA)^+^ stress fibers and the increase in cell size. TFEB-EGFP was present in the nuclei of untreated epicardial cells and was reduced twice in cells differentiated into α-SMA^+^ myofibroblasts.

**Fig. 2:**
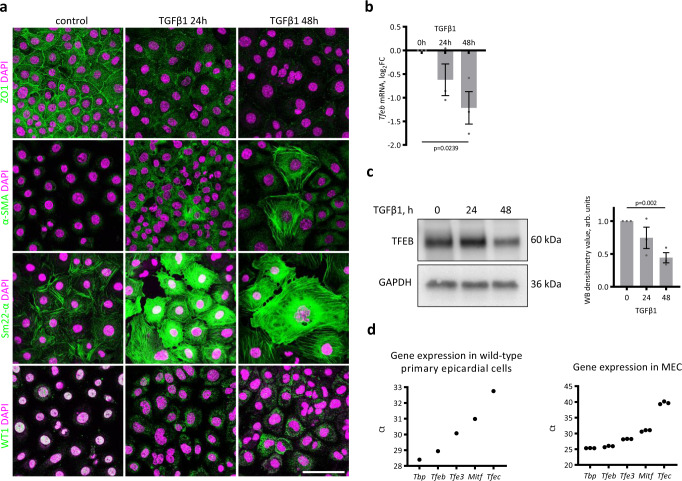
*Tfeb* is downregulated during EMT in MECs. **a** MECs undergo EMT upon TGFβ1 treatment. MECs were treated with 10 ng/ml TGFβ1 in 2% FCS for 24 and 48 h to trigger EMT and then fixed and immunostained for epithelial (ZO1), epicardial (WT1), and SMC (αSMA and Sm22α) markers. The experiment was repeated three times with similar results. The scale bar is 100 µm. **b**, **c** *Tfeb* is downregulated during EMT induced by TGFβ1 treatment in MECs, as demonstrated by real-time PCR analysis (**b**) and western blots (**c** left; **c** right: densitometric analysis of western blots). Values are shown as the mean ± SEM of three independent replicates. Student’s *t* two-tailed test *p* values are reported in the plots. **d** *Tfeb* is the most highly expressed member of the MITF family in epicardial cells. Real-time PCR analysis of gene expression in wild-type primary epicardial cells (left) and MECs (right). A pool of six explants was used in the case of primary cells, and three independent experiments were performed in the case of MECs. Source data are provided as a Source Data file.

To implement a more suitable model to study TFEB functions, we derived a mouse embryonic epicardial cell line (MEC), as described in ref. 47. MECs showed a typical epicardial cobblestone-like morphology (Supplementary Fig. 3a) with cortical distribution of the tight junction protein ZO1 (Fig. 2a), expressed the epicardium-specific TFs WT1 and TBX18 and did not contain contaminating cells, such as endothelial cells (ECs; CD31^+^), cardiomyocytes (cTnT^+^), fibroblasts (PDGFRα^+^), and SMCs (PDGFRβ^+^; Supplementary Fig. 3b). TGFβ1 treatment triggered EMT, as inferred by the increase in cell size, the loss of WT1 and ZO1 and the positivity for the smooth muscle proteins αSMA and Sm22α (Fig. 2a). MECs expressed TFEB, but during EMT, both TFEB transcript and protein levels gradually decreased (Fig. 2b, c). Both in primary epicardial cells and in MECs, *Tfeb* was the most highly expressed member of the MITF family (Fig. 2d).

### *Tfeb* overexpression in the embryonic epicardium is lethal due to an impairment of EMT

*Tfeb* downregulation in epicardial cells during EMT suggested its role in EMT regulation. To investigate this possibility, we generated a mouse model in which *Tfeb* expression in EPDCs persisted.

**Fig. 3:**
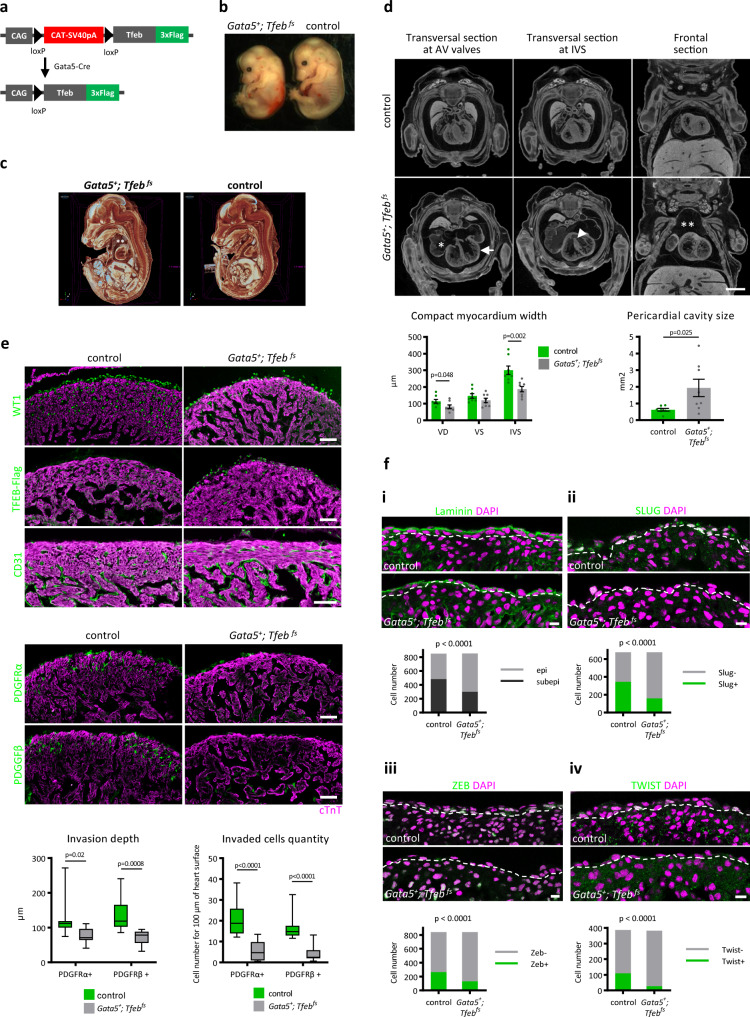
*Tfeb* overexpression in the epicardium is lethal due to inhibited EMT and fibroblast and SMC differentiation. **a** Scheme of the transgenic allele used for the creation of the *Tfeb-*overexpressing mice. The mouse *Tfeb* gene coding sequence, combined with a 3xFlag tag, was inserted under the control of the chicken CAG promoter and floxed stop cassette. **b** Microscopy images of E15.5 *Gata5*^*+*^*; Tfeb* ^*fs*^ and control embryos. **c**, **d** E15.5 *Gata5*^*+*^*; Tfeb* ^*fs*^ embryos had a thinner myocardium and enlarged pericardial cavity. Micro-CT scans (**d**, top) and 3D reconstruction (**c**) of E15.5 *Gata5*^*+*^*; Tfeb* ^*fs*^ and control embryos and quantification of the morphological defects (**d**, bottom). *Gata5*^*+*^*; Tfeb* ^*fs*^ embryos are oedemic (*marks enlarged atria, **indicates the increased area of pericardiac sac), with a thinner compact myocardium layer measured in the right (VD) and left (VS) ventricles and at intraventricular septum (IVS) (arrow). Six of eight mutant embryos showed an incomplete ventricular septum (arrowhead). Scale bar is 1 mm. Quantification of the compact myocardium width appearing in a transversal micro-CT section at valve height (left) and of the pericardial cavity area appearing in the frontal micro-CT section (right). The phenotypic defects of 8 embryos were quantified using Bruker Micro-CT DataViewer software. Values are shown as the mean ± SEM (Student’s two-tailed *t* test *p* values are reported in the plots). **e** E15.5 *Gata5*^*+*^*; Tfeb* ^*fs*^ embryos showed a severe reduction in SMCs and fibroblasts in myocardial tissue. Immunofluorescence analysis for WT1, TFEB-FLAG, CD31, PDGFRα, PDGFRβ (green), and cTnT (magenta) in E15.5 *Gata5*^*+*^*; Tfeb* ^*fs*^ and control embryos. The scale bar is 50 μm. Quantification of the average invasion depth of PDGFRα- and PDGFRβ-positive cells in the compact myocardium (bottom left); quantification of the number of invaded PDGFRα- and PDGFRβ-positive cells, normalized to 100 µm of heart surface (bottom right), in a microscopy image. Six embryos of each genotype and two images for each embryo were quantified with ImageJ software. Box plots show the quartiles, the 5th and 95th percentiles (whiskers). Student’s two-tailed *t* test *p* values are reported in the plots. **f** E13.5 *Gata5*^*+*^*; Tfeb* ^*fs*^ embryos demonstrated inhibited epicardial cell delamination (i) and a reduction in the expression of the key EMT TFs Slug (ii), Zeb (iii), and Twist (iv). Immunofluorescence analysis for laminin, Slug, Zeb, Twist (green), and nuclei (magenta) in E13.5 *Gata5*^*+*^*; Tfeb* ^*fs*^ and control embryos. The overlay of laminin, Slug, Zeb, Twist, and cTnT immunostainings is shown in Supplementary Fig. 5. The dashed line shows the myocardium surface. The scale bar is 10 μm. Nuclei were counted in ImageJ (5 embryos of each genotype, at least 5 images for embryo) as follows. i Quantification of epithelial cell nuclei (localized above immunostaining for laminin basement membrane) and delaminated subepicardial EPDC nuclei (localized under the basement membrane). ii–iv Quantification of epicardial and subepicardial cell nuclei positive for EMT-regulating TFs. Source data are provided as a Source Data file.

*Tfeb-flag*^*fs*^ mice^48^ were crossed with mice expressing *Cre* under the control of the epicardium-specific *Gata5* promoter^8^ (Fig. 3a). *Tfeb* overexpression in the epicardium was lethal. At weaning, the analysis of 54 animals showed the absence of *Gata5*^*+*^*; Tfeb*^*fs*^ mice in contrast with the 25% expected Mendelian frequency, given that the parents were heterozygous for *Gata5* and *Tfeb*^*fs*^ alleles (*χ*^2^ test; *p* value <0.001; Table 1). To establish when exactly the embryos died, we collected litters at different gestational stages. At E17.5, among the 29 collected embryos, no live *Gata5*^*+*^*; Tfeb*^*fs*^ embryos were found (*χ*^2^ test; *p* value <0.05; Table 1); however, 2 of the 3 collected resorbed embryos had the *Gata5*^*+*^*; Tfeb*^*fs*^ genotype. The distribution of genotypes of the embryos collected before E15.5 was not different from the expected distribution (*p* value >0.99).

**Table 1:** *Tfeb* overexpression in the epicardium is lethal

	Total	*Gata5*^*+*^	*Tfeb*^*fs*^	Wild-type	*Gata5*^*+*^*; Tfeb*^*fs*^
Expected	100%	25%	25%	25%	25%
Stage					
At weaning	54	15 (27.8%)	18 (33.3%)	21 (38.9%)	0 (0%)**
At E17.5	29	10 (34.5%)	9 (31.0%)	10 (34.5%)	0 (0%)*
Before E15.5	122	29 (23.8%)	31 (25.4%)	31 (25.4%)	31 (25.4%)

Genotypes of offspring from *Gata5*^+^ and *Tfeb*^*fs*^ crosses. At E17.5, three resorbed embryos were found, two of which had the *Gata5*^+^; *Tfeb*^*fs*^ genotype. The segregation ratio was analyzed with the *χ*^2^ test against the expected Mendelian ratio of 1:1:1:1. *p < 0.05, **p < 0.001

*Gata5*^*+*^*; Tfeb*^*fs*^ embryos at E15.5 were smaller than their wild-type littermates, with hemorrhagic areas (Fig. 3b). Microcomputed tomography (Micro-CT) scanning (Fig. 3c, d) revealed that the *Tfeb*-overexpressing embryos were edematous, as indicated by the significant increase in pericardial cavity volume, and demonstrated enlarged atria. Mutant mice also had a thinner compact myocardium layer, with a significant decrease in thickness in the right ventricle and interventricular septum. Six of eight analyzed *Gata5*^*+*^*; Tfeb*^*fs*^ embryos developed an incomplete interventricular septum, while the control littermates (*n* = 8) did not show any defects. Consistent with the fact that Gata5 is active in the PE at E9.5–10.5^8^, Cre recombinase and TFEB-FLAG were already detected in WT1^+^ cells at E9.5 (Supplementary Fig. 4). Immunostaining of E15.5 hearts (Fig. 3e) showed that the epicardial layer of mutant embryos, marked with WT1, was fully formed and expressed TFEB-FLAG, indicating that *Tfeb* overexpression did not alter the survival or proliferation of epicardial cells. However, many fewer WT1-positive cells were found inside the myocardium of the *Gata5*^*+*^*; Tfeb*^*fs*^ embryos (Fig. 3e). Moreover, PDGFRβ^+^ and PDGFRα^+^ cells, which are both derived from epicardial cells, were strikingly decreased in the mutant hearts (Fig. 3e). The quantification of both the number of PDGFRα^+^ or PDGFRβ^+^ cells invading the myocardium and the depth of the invasion of each PDGFRα^+^ or PDGFRβ^+^ cell from the surface confirmed the severe phenotype (Fig. 3e). Taking all these observations together, we hypothesized that *Tfeb* overexpression would halt the differentiation of EPDCs, leading to the development of hearts with defects derived from the poor differentiation of epicardial cells in vSMCs and fibroblasts. Of note, the CD31^+^ endothelial cell population was not diminished in the *Gata5*^*+*^*; Tfeb*^*fs*^ myocardium (Fig. 3e), according to previous findings that the coronary endothelium mainly derives from the endocardium and sinus venosus^49,50^. To verify whether *Tfeb* overexpression influenced epicardial EMT or the subsequent migration and differentiation of EPDCs, we analyzed the *Gata5*^*+*^*; Tfeb*^*fs*^ embryos at E13.5, when EMT has already begun^16^. To visualize epicardial cell delamination, we immunostained the basement membrane with an antibody against laminin and counted epicardial cell nuclei localized above the membrane and subepicardial cell nuclei between the basement membrane and the myocardium surface (Fig. 3f i). In the control embryos, the ratio between epicardial and subepicardial cells was 1:1.31, while in the *Gata5*^*+*^*; Tfeb*^*fs*^ embryos, it was 1:0,54, suggesting a strong inhibition of derivation of mesenchymal cells (Fig. 3f i). We also quantified the epicardial cells and EPDCs positive for the EMT-activating TFs Slug, Zeb and Twist^51^. These EMT markers were consistently reduced in the *Gata5*^*+*^*; Tfeb*^*fs*^ embryos: from 51% of Slug^+^ cells in the controls to 24% in the *Tfeb*-overexpressing epicardial cells and EPDCs, from 31% to 16% for Zeb^+^ and from 28% to 7% for Twist^+^ (Fig. 3f ii–iv). No difference in proliferation rate, evaluated as positivity for Ki67, was observed (Supplementary Fig. 5v). These data suggest that *Tfeb* overexpression severely inhibited, but did not fully abolish, epicardial EMT starting from the early stages.

### TFEB regulates EMT in primary mouse epicardial cells and MECs

**Fig. 4:**
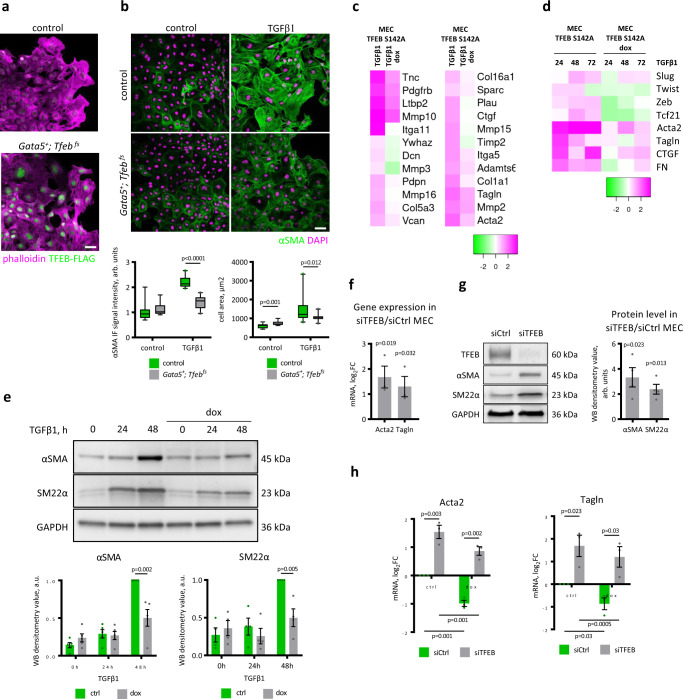
TFEB regulates EMT in primary mouse epicardial cells and MECs. **a**, **b** *Tfeb* overexpression inhibits myofibroblast differentiation in primary mouse epicardial cells. **a** Epicardial explants from *Gata5*^*+*^*; Tfeb* ^*fs*^ embryos expressed TFEB-FLAG. Hearts of E11.5 control and mutant embryos were cultured in DMEM with 10% FCS for 24 h, hearts were removed, and explants were cultured for 24 h in DMEM with 10% FCS. Immunostaining with anti-Flag antibody (green) and phalloidin (magenta). Scale bar 50 µm. **b** Epicardial explants from *Gata5*^*+*^*; Tfeb* ^*fs*^ embryos show a weaker response to TGFβ1. Images of control and *Gata5*^*+*^*; Tfeb* ^*fs*^ epicardial explants challenged with 10 ng/ml TGFβ1 for 48 h and immunostained for αSMA and nuclei (DAPI). Scale bar 50 µm. αSMA immunofluorescence signal intensity (left plot) and cell area (right plot) were quantified with ImageJ software using 3–6 explants for experimental conditions and at least five images for an explant. Box plots show the quartiles, the 5th and 95th percentiles (whiskers). Student’s two-tailed *t* test *p* values are reported in the plots. **c**–**e** TFEB S142A overexpression in MECs inhibits myofibroblast differentiation induced by TGFβ1. **c** Heatmap of gene expression, analyzed by real-time PCR, in the MEC-TFEB S142A and doxycycline-treated MEC-TFEB S142A cells treated with TGFβ1 for 24 h (values are log_2_FC, 0 value was attributed to MEC-TFEB S142A cells not treated with TGFβ1). Only differentially expressed genes (Student’s test *p* value <0.05) between the doxycycline-induced and noninduced cells are shown. **d** Real-time PCR analysis of the expression of epithelial- and mesenchymal-specific genes in the MEC-TFEB S142A cells, where TFEB S142A expression was triggered with doxycycline, treated with TGFβ1 for 24, 48, and 72 h. Gene expression is shown in the heatmap as log_2_FC, 0 value was attributed to MEC-TFEB S142A cells not treated with TGFβ1. **e** TFEB S142A overexpression activated by doxycycline (dox) inhibits the upregulation of αSMA and SM22α proteins induced in the MEC-TFEB S142A cells by TGFβ1 treatment for 24 h and 48 h, western blot analysis, top. Densitometric analysis of western blots, bottom. Values are shown as the mean ± SEM, *n* = 5 (αSMA), 4 (SM22α). Student’s two-tailed *t* test *p* values are reported in the plots. **f**, **g** *Tfeb* silencing induces EMT in MECs. **f** Real-time PCR analysis of SMC (*Acta2* and *Tagln*) markers in *Tfeb*-silenced MECs. Values are shown as the mean ± SEM, *n* = 3. Student’s two-tailed *t* test *p* values are reported in the plot. ***g*** Western blot analysis for αSMA and SM22α in *Tfeb*-silenced MECs (left), densitometric analysis of western blots (right). Values are shown as the fold change between siTFEB and siCtrl MECs, average ± SEM, *n* = 4. Student’s two-tailed *t* test *p* values are reported in the plot. **h** TFEB S142A overexpression rescues the upregulation of mesenchymal markers in *Tfeb*-silenced MEC-TFEB S142A cells. Real-time PCR analysis of *Acta2* and *Tagln* showed that their expression was upregulated in *Tfeb*-silenced MEC-TFEB S142 cells and restored after overexpressing TFEB S142A by doxycycline treatment (dox). Values are shown as the mean ± SEM, *n* = 3. Student’s two-tailed test *p* values are shown in the plots. Source data are provided as a Source Data file.

Thereafter, we evaluated the effect of *Tfeb* overexpression on TGFβ1-induced EMT in primary epicardial cells isolated from the *Gata5*^*+*^*; Tfeb*^*fs*^ mice. Epicardial explants from the mutant embryos grew similarly to those from the wild-type embryos and contained TFEB-FLAG in the nuclei (Fig. 4a), indicating that overexpressed TFEB did not affect cell survival and proliferation. However, the *Gata5*^*+*^*; Tfeb*^*fs*^ epicardial cells showed a significantly weaker response to TGFβ1 treatment, as demonstrated by αSMA expression and cell size (Fig. 4b).

Superimposable results were obtained by infecting MECs with a lentivirus encoding the constitutively active TFEB mutant S142A^26^ under the control of a doxycycline-inducible promoter (MEC-TFEB S142A) (Supplementary Fig. 6a, b). After induction, TFEB S142A was localized to nuclei, ensuring protein activity (Supplementary Fig. 6b), and after 2 days, it did not show toxic effects, as demonstrated by a slight increase in BrdU incorporation, consistent with previous reports^33,34^ (Supplementary Fig. 6c).

To further support the observation that TFEB S142A overexpression inhibited MEC response to TGFβ1, we analyzed the expression of 85 EMT-related genes, such as myofibroblast-specific proteins and extracellular matrix components, selected by Gene Ontology annotations, in the MECs overexpressing TFEB S142A and treated with TGFβ1 by real-time PCR. Twenty-five genes were significantly downregulated in the doxycycline-treated MEC-TFEB S142A cells stimulated with TGFβ1 compared to the TGFβ1-stimulated MEC-TFEB S142A cells nontreated with doxycycline (Student’s test *p* value <0.05; Fig. 4c). Next, we analyzed the expression of well-known EMT markers in a time-course experiment after TGFβ1 stimulation (Fig. 4d). *Tfeb* overexpression blocked the induction of the EMT-activating TFs *Twist*, *Zeb* and *Tcf21*, and delayed and diminished the increase in the transcription factor *Slug*, the mesenchymal markers *CTGF* and *FN* and the smooth muscle markers *Acta2* and *Tagln*. This analysis was further validated by evaluating the protein levels of selected targets. Figure 4e shows that the increased expression of the *Acta2* gene-encoded α-SMA and *Tagln* gene-encoded Sm22α proteins induced by TGFβ1 was blunted in the doxycycline-treated MEC-TFEB S142A cells.

Since overexpressed TFEBS142A acted as an inhibitor of EMT in MEC cells, siRNA-mediated silencing was used to determine whether endogenous TFEB might have the same role (Supplementary Fig. 6d). We evaluated the expression of SMC markers (α-SMA and Sm22α) at the mRNA (Fig. 4f) and protein (Fig. 4g) levels and found that in the *Tfeb*-silenced MECs, the SMC markers were upregulated. The expression of TFEB S142A after *Tfeb* silencing rescued the MEC phenotype (Fig. 4h and Supplementary Fig. 6e). Independent of the presence of TGFβ1, *Tfeb*-silenced MECs showed an upregulation of *Acta2* and *Tagln* transcripts, which was reduced after TFEB S142A reintroduction (Fig. 4h).

### TFEB modulates EMT through TGIF1

To provide insights into the mechanisms sustaining the regulatory effect of TFEB in the mesenchymal transition of epicardial cells, we performed chromatin immunoprecipitation sequencing (ChIP-seq) in MECs overexpressing TFEB S142A. To prevent the possible side effects of excessive TFEB accumulation, we set 8 h of doxycycline induction as the minimum to guarantee TFEB S142A overexpression in most of the nuclei (Supplementary Fig. 6g). The 8258 peaks of TFEB S142A DNA binding were called with medium stringency (Supplementary Fig. 7a). The annotation of peak genomic distribution executed by HOMER software^52^ showed that gene promoters and 5’ untranslated regions (UTRs) were strongly enriched among the TFEB target regions (Supplementary Fig. 7b). For further analysis, we focused on the set of 1899 peaks superimposing mouse promoter regions, defined as −2500 bp and +2500 bp from the transcription start site (TSS), of 1928 protein-coding genes, annotated by GREAT 3.0.0 software^53^. Thirty-two percent of the TSS-associated peaks directly contained a TFEB-binding motif, specified by the JASPAR matrix (relative score >0.6)^54^. Furthermore, de novo motif discovery on the complete set of sequences of the TSS-associated peaks demonstrated a strong enrichment in the E-box CACGTG TFEB-binding motif (*p* value 10^−29^; Supplementary Fig. 7a). Functional annotation of TFEB target genes performed by GREAT (Supplementary Fig. 7c) revealed the most important enrichment for functional groups related to the regulation of transcription, including DNA binding and TF activity itself as well as TF binding and cofactor activity. In terms of biology, TFEB targets are involved in the regulation of cell differentiation, epithelial phenotype, cytoskeleton rearrangement, adhesion, and motility, which are the processes underlying EMT. Notably, among TFEB target genes, there were components of the TGFβ/SMAD and PDGFR signaling pathways, which are involved in epicardial EMT regulation.

**Fig. 5:**
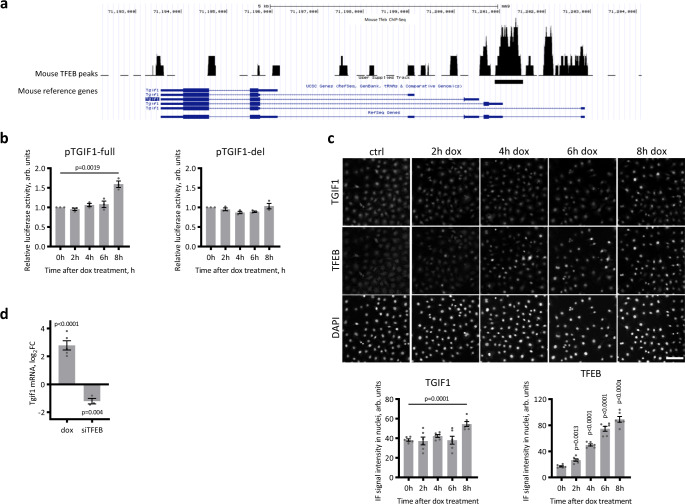
TGIF1, the SMAD transcriptional corepressor, is a target of TFEB. **a** Visualization of the peak of TFEB binding in the region of the TGIF1 promoter in the UCSC genome browser and ChIP-seq analysis data for doxycycline-treated MEC-TFEB S142A cells. **b** Analysis of TFEB regulation of the *Tgif1* promoter. Luciferase activity was measured in pTGIF1-full- and pTGIF1-del-transfected MEC-TFEB S142A cells, where TFEB S142A expression was induced by doxycycline for 2–8 h. Luciferase activity in each of 3 replicate experiments was normalized to that in noninduced cells. Values are shown as the mean ± SEM. Student’s two-tailed *t* test *p* value is shown in the plot. **c** Immunofluorescence analysis (top) and quantification (bottom) of TGIF1 and TFEB S142A in the nuclei of MEC-TFEB S142A cells, where TFEB S142A expression was induced with doxycycline for 2–8 h. The scale bar is 100 µm. Six microscopy images were quantified for an experimental point, and the values are shown as the average ±SEM. Student’s two-tailed *t* test was performed between each time point of dox treatment and the untreated sample, *p* values are reported in the plot. **d** Real-time PCR analysis of *Tgif1* mRNA in MECs overexpressing TFEB S142A for 24 h (left) or silenced for *Tfeb* (right). Values are shown as the mean ± SEM, *n* = 5 for the TFEB S142A overexpression experiment, *n* = 3 in the *Tfeb* silencing experiment. Student’s two-tailed *t* test *p* values are reported in the plot). Source data are provided as a Source Data file.

We demonstrated above that the TFEB inhibitory effect on EMT was underpinned by the decreased expression of many genes. The presence of many transcriptional regulators and a marked enrichment of genes belonging to the TGFβ pathway in the ChIP-seq dataset prompted us to hypothesize that TFEB might directly upregulate a transcriptional repressor or an inhibitory cofactor of the TGFβ-activated gene response, which is crucial for epicardial EMT. Among the TFEB ChIP-seq targets harboring this function, the *Tgif1*, *Ski*, and *Skil* genes were identified^45,55,56^ (Fig. 5a and Supplementary Fig. 8a, b). We excluded *Ski* and *Skil* after experimental validation. In MECs, *Ski* expression did not depend on overexpressed or endogenous TFEB (Supplementary Fig. 8c). *Skil* mRNA expression was upregulated by TFEB S142A and downregulated by *Tfeb* silencing (Supplementary Fig. 8d); nevertheless, *Skil* silencing did not rescue the inhibition of the *Acta2* transcript by TFEB S142A overexpression (Supplementary Fig. 8e, f).

**Fig. 6:**
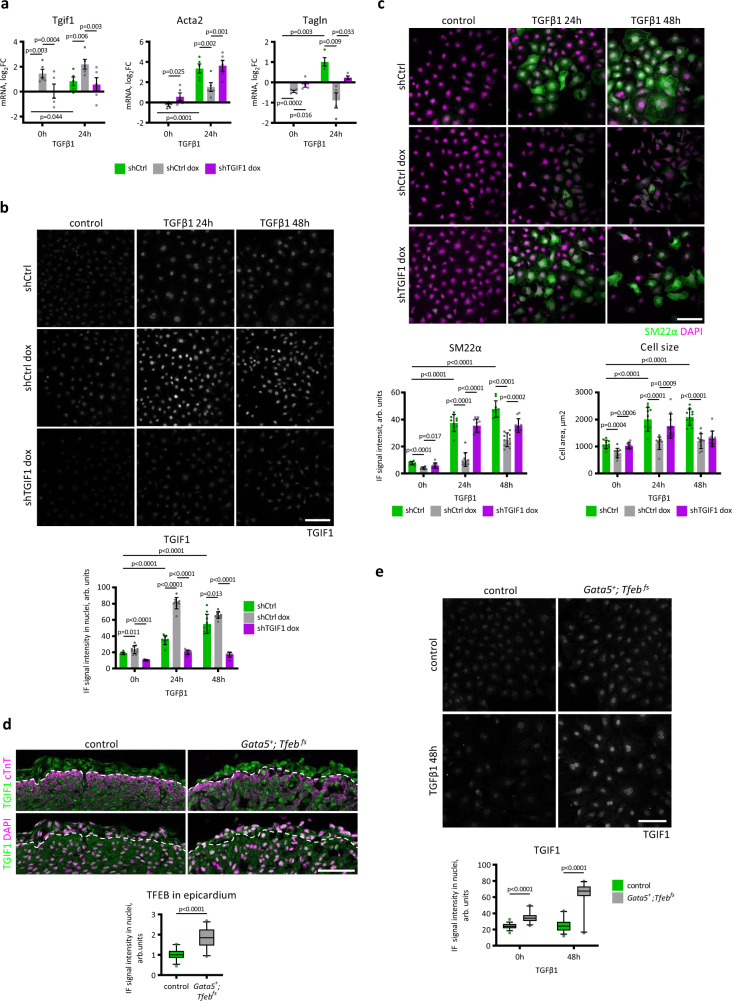
The inhibitory effect of *Tfeb* overexpression on TGFβ-induced EMT is mediated by TGIF1. **a** *Tgif1* silencing rescues TFEB-dependent inhibition of myofibroblast differentiation in MEC-S142A cells in response to TGFβ1. Real-time PCR analysis of MEC-TFEB S142A cells infected with lentiviruses encoding shTGIF1 or shCtrl, where TFEB S142A overexpression was induced with doxycycline, and treated with TGFβ1 for 24 h. Values are shown as the mean ± SEM, *n* = 5 (*Tgif1, Acta2*), *n* = 4 (*Tagln*). Student’s two-tailed test *p* values are reported in the plots. **b**, **c** *Tgif1* silencing rescues TFEB-dependent inhibition of myofibroblast differentiation in MEC-S142A cells in response to TGFβ1. Immunostaining for TGIF1 (**b**) and Sm22α (**c**) in MEC-S142A cells infected with lentiviruses encoding shTGIF1 or shCtrl, where TFEB S142A overexpression was induced with doxycycline, and treated with TGFβ1 for 24 h and 48 h. The immunofluorescence signal was quantified (plots in bottom) in all cells for Sm22α and in nuclei selected with DAPI for TGIF1 by ImageJ software (10 images for a sample). Cell area was measured by the background signal of the anti-Sm22α antibody. Values are shown as the mean ± SEM. Student’s two-tailed test *p* values are reported in the plots. The scale bar is 100 μm. **d**, **e** Validation in vivo: *Tgif1* is upregulated in the epicardium of *Tfeb*-overexpressing mice. **d** Immunostaining for TGIF1 and cTnT; nuclei are stained with DAPI in *Gata5*^*+*^*; Tfeb* ^*fs*^ and control embryos at E15.5. The dashed line indicates the myocardium surface. The TGIF1 immunofluorescence signal was quantified in nuclei selected by DAPI with ImageJ software (bottom). Eight embryos for each genotype and at least three images for an embryo were used for quantification. Box plot shows the quartiles, the 5th and 95th percentiles (whiskers). Student’s two-tailed test *p* < 0.0001 The scale bar is 50 μm. **e** Immunostaining for TGIF1 in epicardial explants of *Gata5*^*+*^*; Tfeb* ^*fs*^ and control embryos treated with TGFβ1 and control. The immunofluorescence signal was quantified in nuclei selected by DAPI with ImageJ software (bottom). Four to six explants were used for each experimental point, and at least 6 images of an explant were used for quantification. Box plot shows the quartiles, the 5th and 95th percentiles (whiskers). Student’s two-tailed test *p* < 0.0001. The scale bar is 100 μm. Source data are provided as a Source Data file.

TGIF1 was reported to inhibit TGFβ signaling by directly binding the SMAD2 and SMAD4 complexes and repressing TGFβ-induced SMAD-mediated transcription^44,45^. ChIP-seq analysis revealed a peak of TFEB S142A binding overlapping the *Tgif1* promoter region (Fig. 5a). Bioinformatic analysis of the sequence of the ChIP-seq peak revealed the presence of a TFEB Jaspar binding site. To demonstrate the direct activity of TFEB on the *Tgif1* promoter, we designed two luciferase reporter constructs with the *Tgif1* full promoter sequence (−1106; +310 bp around the TSS) and the mutant version lacking the TFEB binding peak (pTGIF1-full and pTGIF1-del), which were transfected into MEC-TFEB S142A cells. The overexpression of TFEB S142 by doxycycline resulted in a 60% increase in pTGIF1 full promoter activity, while no increase was observed for the mutated promoter (Fig. 5b). Accordingly, immunofluorescence analysis showed an increase in TGIF1 protein expression in the TFEB S142A-MECs, starting after 8 h of doxycycline treatment (Fig. 5c) and persisting after 24 and 48 h (Fig. 6b). This finding was confirmed by transcript analysis showing that *Tgif1* mRNA expression was strongly upregulated upon TFEB S142A overexpression and downregulated in the *Tfeb*-silenced MECs (Fig. 5d).

To verify whether TFEB inhibited TGFβ1-induced EMT in the TFEB S142A-MECs by upregulating *Tgif1*, we performed a rescue experiment by simultaneous overexpression of TFEB S142A and knockdown of *Tgif1* by a lentiviral vector carrying shTGIF1. After 24 h, the cells were treated with TGFβ1. Doxycycline-induced TFEB S142A expression caused a strong increase in *Tgif1* mRNA (Fig. 6a) and protein (Fig. 6b) expression, which was mainly nuclear. *Tgif1* silencing completely abolished the observed upregulation induced by TFEB S142A; however, importantly, it did not reduce the mRNA and protein levels below those in the untreated cells (Fig. 6a, b). In addition, our experiment showed the induction of TGIF1 by TGFβ1 under normal conditions, confirming previous reports^57^. Fig. 6a shows that *Tgif1* knockdown rescued the inhibitory effect of TFEB S142A on the upregulation of *ACTA2* and *Tagln* mRNA after stimulation with TGFβ1. Similarly, the expression of the *Tagln* gene-encoded Sm22α protein observed in the MECs challenged with TGFβ1 was inhibited by TFEB S142A overexpression but completely rescued by *Tgif1* silencing (Fig. 6c). The increase in cell size, which characterizes EMT, was also controlled by the TFEB-TGIF1 signaling axis. *Tgif1* knockdown abolished the TFEB S142A-induced inhibition of the cell size increase upon TGFβ1 treatment (Fig. 6c). Altogether, the results of the rescue experiment suggested that TFEB inhibited TGFβ1-induced EMT in epicardial cells in vitro by directly upregulating *Tgif1*.

To validate these in vitro findings in vivo, we analyzed the expression of *Tgif1* in the epicardium of the *Gata5; Tfeb*^*fs*^ and control E15.5 embryos by immunofluorescence and found a strong increase in TGIF1 protein expression in the nuclei of the mutant embryos (Fig. 6d). This result was further confirmed in primary epicardial cells cultured from the *Gata5*^*+*^*; Tfeb*^*fs*^ and control embryos. Both under basal conditions and during the differentiation induced by TGFβ1, *Tgif1* was significantly upregulated in the TFEB-overexpressing cells (Fig. 6e).

Altogether, these data indicate that overexpressed *Tfeb* acts through *Tgif1* upregulation, ultimately inhibiting the cell response to TGFβ1 both in vitro and in vivo.

### Dynamics of the TGFβ1 regulation of TGIF1 and TFEB

**Fig. 7:**
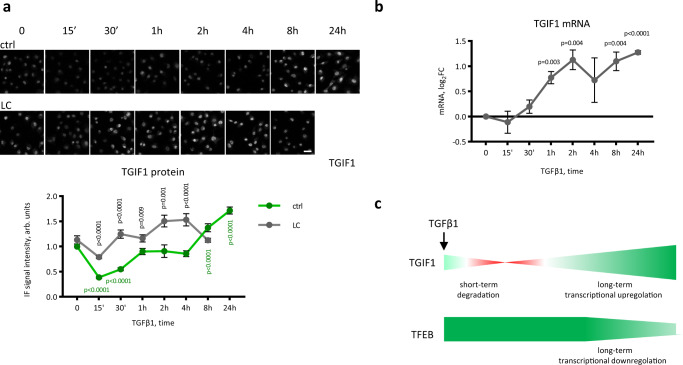
TGFβ1 regulation of TGIF1. **a** TGFβ1 stimulation causes short-term TGIF1 protein degradation and a long-term protein increase. Immunostaining for TGIF1 (top) and its quantification (bottom) in MECs treated with TGFβ1 for the indicated time points in the presence or absence of 50 μM lactacystin (LC), an inhibitor of proteosomes. Scale bar is 50 μm. The experiment was repeated three times, six images for each time-point were used for analysis in each experiment. Values are shown as the average ±SEM. Student’s two-tailed test *p* values are shown as follows: *p* values in black font above the lines are calculated between the control and lactacystin-treated samples; *p* values in green font below the lines are calculated between the nontreated control sample and TGFβ1-treated control samples. **b** Real-time PCR analysis of the *Tgif1* transcript in MECs treated with TGFβ1 for the indicated time points. Values are shown as the mean ± SEM, *n* = 3. Student’s two-tailed test *p* values between TGFβ1-treated samples and the nontreated sample are shown in the plot. **c** A model showing the dynamics of TGIF1 and TFEB regulation by TGFβ1 based on the data of Fig. 7 a, b and Supplementary Fig. 9a. Source data are provided as a Source Data file.

To investigate the physiological role of TFEB in TGIF1 regulation of TGFβ1 signaling, we first examined the time-dependence of TGIF1 expression after TGFβ1 stimulation. For the transcriptional response to TGFβ1 signaling, TGIF1 repression should be promptly lifted. This kind of regulation was shown for other TGFβ1 inhibitors such as Ski and SnoN: after TGFβ1 treatment, the repressor proteins are rapidly degraded by the ubiquitin-proteasome machinery, with the minimum level reached in 15–45 min; at the same time, their mRNA is de novo synthesized, leading to the restored protein level at ∼2 h^56,58–60^. To determine whether TGIF1 shares the same regulatory pattern, we evaluated its mRNA and protein levels in MECs after TGFβ1 treatment in the absence and presence of the proteosome inhibitor lactacystin. TGFβ1 stimulation caused a significant decrease in TGIF1 protein expression after 15 and 30 min, which was followed by a restoration of protein levels after 1 h and an increase after 24 h (Fig. 7a). Lactacystin completely abolished TGIF1 protein level decrease, suggesting the involvement of the ubiquitin-proteasome system degradation (Fig. 7a). A TGIF1 mRNA increase was observed starting from 1 h of TGFβ1 treatment (Fig. 7b). Therefore, it is conceivable that, similar to Ski and SnoN, TGFβ1 caused rapid short-term TGIF1 protein degradation and long-term transcriptional upregulation (Fig. 7c). We also examined whether TGFβ1 had any short-term effects on TFEB protein levels and did not observe any effects (Supplementary Fig. 9a and Fig. 7c).

### The role of the TFEB-TGIF1 axis in TGFβ signaling under physiological conditions

**Fig. 8:**
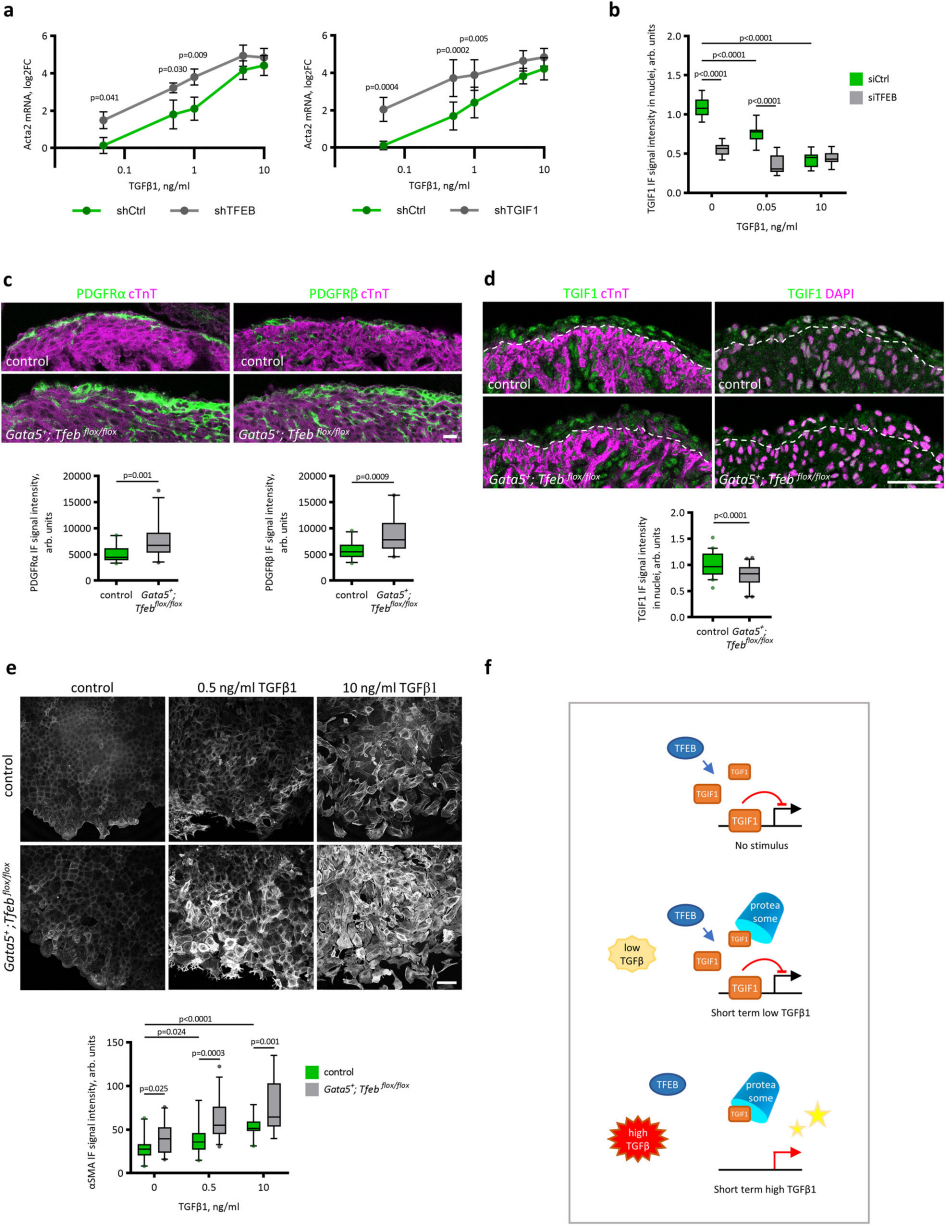
*Tfeb* silencing in MECs sensitized them to lower concentrations of TGFβ1. **a** *Tfeb* and *Tgif1* silencing caused a stronger upregulation of the *Acta2* transcript in response to low doses of TGFβ1. MECs were infected with a lentivirus coding for a shRNA for *Tfeb* or *Tgif1* or control shRNA. Cells were treated with 0.05, 0.5, 1, 5, and 10 ng/ml TGFβ1 for 24 h. The *Acta2* mRNA quantity was assessed by real-time PCR analysis. Values are shown as the mean ± SEM, *n* = 4. Two-way ANOVA Bonferroni correction *p* values are shown in the plots. **b** Effect of *Tfeb* silencing on TGIF1 protein levels in MECs treated with low (0.05 ng/ml) and high (10 ng/ml) doses of TGFβ1 for 15 min. Images are shown in Supplementary Fig. 9b. Experiment was repeated three times, six images for each experimental point were analyzed. Box plot shows the quartiles, the 5th and 95th percentiles (whiskers). Student’s two-tailed test *p* values are shown in the plot. **c** *Tfeb* knock out in epicardium favors premature EPDCs differentiation onto vSMC and fibroblast precursors at E13.5. Immunofluorescence analysis for PDGFRα, PDGFRβ (green) and cTnT (magenta) in E13.5 *Gata5*^*+*^*; Tfeb* ^*flox/flox*^ and control embryos (top) and its quantification (bottom). Scalebar is 10 μm. Five embryos of each genotype, at least four images for embryo were used for quantification in ImageJ. Total immunofluorescence signal of a marker in the image was normalized to the length of the myocardium surface in the image. Box plots show the quartiles, the 5th and 95th percentiles (whiskers). Student’s two-tailed test *p* values are shown in the plots. **d** *Tgif1* is downregulated in the epicardium of *Gata5*^*+*^*; Tfeb* ^*flox/flox*^ mice. Immunostaining for TGIF1 (green) and cTnT (magenta); nuclei are stained with DAPI (magenta) in *Gata5*^*+*^*; Tfeb* ^*flox/flox*^ and control embryos at E12.5 (top) and its quantification (bottom). The dashed line indicates the myocardium surface. The scale bar is 50 μm. The TGIF1 immunofluorescence signal was quantified in nuclei selected by DAPI with ImageJ software. Four embryos for each genotype and at least 10 images for an embryo were used for quantification (graph on the right). Box plot shows the quartiles, the 5th and 95th percentiles (whiskers). Student’s two-tailed *t* test *p* value is reported in the plot. **e** Epicardial explants from *Gata5*^*+*^*; Tfeb* ^*flox/flox*^ embryos produced more αSMA in response to low (0.5 ng/ml) and high (10 ng/ml) concentrations of TGFβ1 than control explants. *Gata5*^*+*^*; Tfeb* ^*flox/flox*^ and control hearts at E11.5 were explanted in DMEM with 10% FCS for 24 h. Then, the hearts were removed, and the explants were cultured for 48 h in DMEM with 10% FCS and 0.5 ng/ml or 10 ng/ml TGFβ1 and immunostained for αSMA (representave images in top panel). The αSMA immunofluorescence signal was quantified in ImageJ software, and five explants and at least four images for an explant were analyzed for an experimental point (plot in bottom panel). Box plot shows the quartiles, the 5th and 95th percentiles (whiskers). Student’s two-tailed *t* test *p* values are reported in the plot. **f** A proposed model for the role of TFEB and TGIF1 in the regulation of TGFβ1 signaling. Under basal conditions, TFEB expressed in epicardial cells is required to establish an appropriate quantity of TGIF1 protein. TGFβ1 stimulation rapidly initiates proteosome-dependent TGIF1 degradation; however, a low concentration of TGFβ1 is not sufficient to remove TGIF1 repression, thus creating a dose-dependent response. Source data are provided as a Source Data file.

Because mechanistic information revealing the function of TFEB was obtained in *Tfeb*-overexpressing models, we evaluated the TFEB-TGIF1 axis under physiological conditions. We found that endogenous TFEB inhibited mesenchymal differentiation of MECs (Fig. 4f, g) and that in the *Tfeb*-silenced MECs, *Tgif1* was downregulated (Fig. 5d). Next, we compared the effects of *Tfeb* and *Tgif1* silencing on the TGFβ1-elicited dose-dependent transcription of the EMT marker gene *Acta2* in MECs (Fig. 8a). As previously reported^44^, *Tgif1* silencing sensitized and amplified the increase in *Acta2* mRNA in response to both low and high doses of TGFβ1 (Fig. 8a). The shCtrl-expressing cells responded to TGFβ1 starting from concentrations of 0.5 ng/ml, while *Tfeb* or *Tgif1* silencing sensitized the cell response to 0.05 ng/ml TGFβ1. The shTFEB and shTGIF1 MECs showed significantly stronger responses to low concentrations (0.05–1 ng/ml) of TGFβ1. According to the interplay between TFEB S142A and TGIF1 demonstrated above (Fig. 6), it is likely that the sensitization effect of *Tfeb* deletion relied on the lack of TGIF1. To test this hypothesis, we evaluated TGIF1 protein levels in the MECs with *Tfeb* silencing treated with low (0.05 ng/ml) and high (10 ng/ml) concentrations of TGFβ1 for 15 min, the time point at which we observed the lowest level of TGIF1 (Fig. 7a). Low amounts of TGFβ1 (0.05 ng/ml) caused a 30% reduction in TGIF1 examined by immunofluorescence analysis (Fig. 8b and Supplementary Fig. 9b), which was not sufficient to remove transcriptional inhibition of *Acta2* (Fig. 8a). In the MECs stimulated with 10 ng/ml TGFβ1, TGIF1 was reduced by 60%, which allowed a transcriptional response (Fig. 8b, a). In the *siTfeb* MECs, the TGIF1 protein level was low under basal conditions, and 0.05 ng/ml TGFβ1 treatment resulted in a 60% reduction, permitting a transcriptional response (Fig. 8b, a).

To verify in vivo whether the lack of *Tfeb* had the same effects observed in vitro, we generated a mouse model in which *Tfeb* was knocked out in epicardial cells by crossing *Gata5-Cre* with *Tfeb* ^*flox/flo*x^ mice. The resulting *Gata5*^*+*^*; Tfeb* ^*flox/flox*^ mice were healthy and did not present visible pre- or postnatal morphological defects. No difference in the EMT intensity, assessed as the number of delaminated mesenchymal cells and Slug+ epicardial cells and EPDCs, was found in the E13.5 embryos (Supplementary Fig. 10). However, the staining for PDGFRα and PDGFRβ revealed a significant increase in fibroblast and SMC precursors at E13.5 (Fig. 8c). These findings suggest a premature differentiation of the *Tfeb-*deleted EPDCs into SMCs and fibroblasts, which, however, was recovered at later developmental stages.

We analyzed TGIF1 protein levels in epicardial cell nuclei of the E12.5 *Gata5*^*+*^; *Tfeb*^*flox/flox*^ embryos and found a 21% reduction compared to the controls (Fig. 8d). Therefore, we investigated the response to TGFβ1 in epicardial cells cultured from the *Gata5*^*+*^; *Tfeb*^*flox/flox*^ embryos. The efficiency of *Tfeb* deletion in epicardial cells was confirmed by real-time PCR analysis of *Tfeb* mRNA in the mutant and control epicardial explants (Supplementary Fig. 10a). Next, we cultured the *Gata5*^*+*^; *Tfeb*^*flox/flox*^ epicardial explants for 48 h in the presence of 0.5 or 10 ng/ml TGFβ1. Immunostaining revealed that low TGFβ1 amounts significantly increased the synthesis of α-SMA in epicardial cells isolated from the *Tfeb* knockout but not from the control animals (Fig. 8e).

Taken together, these findings suggest a model (Fig. 8f) in which TFEB present in epicardial cells under physiological conditions is necessary to establish a TGIF1 protein quantity threshold, allowing a dose-response to TGFβ1. TGFβ1 stimulation initiates rapid TGIF1 proteasomal degradation; however, a high dose of TGFβ1 is required to reduce TGIF1 to a level that allows initiation of transcription. Long-term TGFβ1 treatment promotes an increase in TGIF1 protein, probably functioning as a feedback mechanism.

It is likely that excess TGIF1 levels observed in the TFEBS142A-overexpressing MECs and in *Gata5*^*+*^*; Tfeb*^*fs*^ embryos strongly inhibited the response even to high doses of TGFβ1. In contrast, an insufficient level of TGIF1 observed in *siTfeb* MECs and in *Gata5*^*+*^; *Tfeb*^*flox/flox*^ primary epicardial cells, allowed the response to low doses of TGFβ1, which under physiological conditions do not promote a response.

### TFEB regulates EMT in vascular endothelial cells and in epithelial MDCK cells

**Fig. 9:**
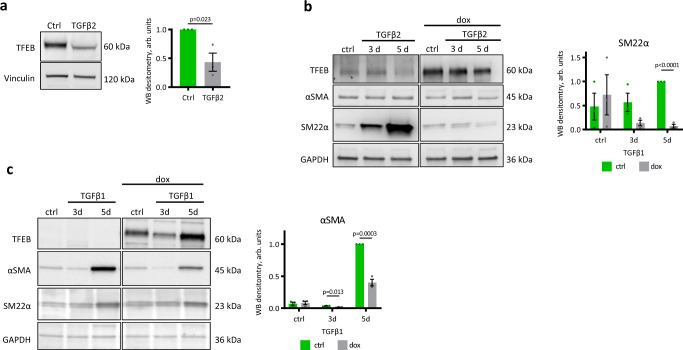
TFEB S142A overexpression inhibits EndMT in human endothelial cells and EMT in MDCK cells. **a** *TFEB* expression decreases during EndMT. HUVECs were treated for 5 days with TGFβ2, and western blotting for TFEB was performed (left). Densitometric analysis of Western blots (right). Values are shown as the mean ± SEM, *n* = 3, Student’s two-tailed *t* test *p* value is reported in the plot. **b** TFEB S142A overexpression inhibits EndMT induced by TGFβ2 in ECs. TFEB S142A expression was induced by doxycycline in EC-TFEB S142A cells, and then, the cells were treated with TGFβ2 for 3 or 5 days. Western blotting for SMC markers (αSMA, SM22α) was performed (left). Membrane stained with anti-TFEB antibody was exposed for different times for the left and right image, given the abundancy of overexpressed TFEB S142A. Densitometric analysis of western blots, right. Values are shown as the mean ± SEM, *n* = 3, Student’s two-tailed *t* test *p* value is reported in the plot. **c** TFEB S142A overexpression inhibits EMT induced by TGFβ1 in MDCK cells. TFEB S142A expression was induced by doxycycline in MDCK-TFEB S142A cells, and then, the cells were treated with TGFβ1 for 3 or 5 days. Western blotting for SMC (αSMA, SM22α) markers was performed (left). Densitometric analysis of western blots (right). Values are shown as the mean ± SEM, *n* = 3, Student’s two-tailed *t* test *p* values are reported in the plot. Source data are provided as a Source Data file.

To verify whether the regulatory effect of TFEB on EMT is restricted to epicardial development or may represent a more general mechanism, we investigated whether this function of TFEB occurs in other cell types. ECs physiologically express *TFEB*, both in embryos and adults^33^, and undergo a process analogous to EMT, named endothelial-to-mesenchymal transition (EndMT). EndMT is governed by a network of growth factors shared with EMT, including the TGFβ family. However, TGFβ2 is considered to be a stronger EndMT inducer than TGFβ1^61,62^. TFEB was expressed in human umbilical vein endothelial cells (HUVECs) under basal conditions and downregulated by TGFβ2 treatment, similar to what we observed in epicardial cells (Fig. 9a). TGFβ2 stimulation for 3 and 5 days triggered EndMT, as demonstrated by the increase in SM22α expression, while αSMA expression did not change significantly. TFEB S142A overexpression completely abrogated the upregulation of SM22α, indicating the inhibition of EndMT (Fig. 9b).

Finally, we investigated the effect of TFEB on TGFβ-induced EMT in renal tubular epithelial cells (MDCK), a prototypical cell model of EMT^63^ (Fig. 9c). TGFβ1 treatment for 5 days induced EMT, characterized by an increase in αSMA and SM22α, and TFEB S142A overexpression strongly diminished αSMA expression. No effect of TFEB S142A on SM22α expression was observed in this cell type, probably because it did not respond strongly to TGFβ in the first place.

Altogether, this evidence supports the idea that the regulatory effect of TFEB on EMT is not confined to epicardial development but is a more general mechanism.

## Discussion

Epicardial EMT is a tightly controlled process. Many TFs are known to act as EMT activators, but the negative regulation of EMT has rarely been investigated. In this study, we demonstrate that the *Tfeb* TF inhibits TGFβ-driven epicardial EMT and EPDC differentiation and invasion during heart development. *Tfeb* is expressed in mouse embryonic epicardium, is downregulated in EPDCs, and can inhibit EMT and the differentiation of epicardial cells into fibroblasts and vSMCs when overexpressed in a mouse model under the control of epicardium-specific Gata5 promoter. Experiments in primary epicardial cells and MECs confirmed that overexpressed TFEB specifically inhibits TGFβ-induced EMT, and conversely, *Tfeb*-silenced cells are both prone to EMT without any additional stimuli and are sensitized to low doses of TGFβ1. *Tfeb* knockout in the epicardium did not influence EMT but favored premature differentiation and invasion of EPDCs, which, however, recovered to control levels at later developmental stages. ChIP-seq analysis and luciferase reporter experiments revealed that the SMAD corepressor TGIF1^44^ is a direct TFEB target. Knockdown and rescue experiments in MECs demonstrated that the inhibitory effect of TFEB on TGFβ1 signaling is mediated by TGIF1. In mouse models in which *Tfeb* was overexpressed or deleted in the epicardium, TGIF1 levels were upregulated or downregulated, respectively, in the same cells, suggesting the TFEB-TGIF1 axis involvement in the regulation of epicardium biology in vivo.

The role of TFEB in the differentiation of cell lineages has already been reported, and while in some reports, it was mediated by canonical autophagy and lysosome activation-related pathways^41–43^, in others, it was independent. Indeed, in osteoblasts, TFEB induced transcriptional downregulation of ATF4 and CHOP TFs, which are important for osteoblast differentiation^40^. Similarly, in liver pluripotent cells, TFEB directly upregulates the transcription of the SOX9 TF, which drives the fate of liver precursors^34^.

*Tfeb* is known to be expressed in a variety of tissues and developmental stages;^64^ however, its robust and specific expression in the developing epicardium has not been investigated to date. *Tfeb* is expressed in epicardial cells from the early proepicardial stage up to E15.5 and becomes undetectable in the adult epicardium. This expression pattern is similar to that reported for the Tbx18^65^ and WT1^66^ TFs. However, in contrast to Tbx18 and WT1, *Tfeb* is completely downregulated when epicardial cells undergo EMT and migrate in the subepicardial space, and it acts as an EMT inhibitor rather than an activator.

*Tfeb* overexpression in *Gata5*^*+*^*; Tfeb*^*fs*^ epicardium led to a typical lethal phenotype, which was described in many mouse models of loss-of-function of TFs or signaling components belonging to the EMT program^8,11,12,18,21,23,67^. The disrupted EMT and, as a consequence, the lack of mural cells covering coronary vessels are thought to be the cause of defective coronary circulation leading to impaired heart function. The hypoplastic myocardium often observed in these models is explained by the well-documented requirement of epicardium-myocardium crosstalk for proper myocardial development^68^. In particular, the epicardial deletion of the TGFβ receptor *Alk5* resulted in a phenotype very similar to that of the *Gata5*^*+*^*; Tfeb*^*fs*^ mice, characterized by thin myocardium, reduced coverage of coronary vessels with vSMCs and inability of primary mutant epicardial cells in culture to respond to TGFβ treatment^69^. To our knowledge, the interventricular septum defect observed in the *Gata5*^*+*^*; Tfeb*^*fs*^ embryos has never been described in any model of epicardium-specific gene targeting. However, it was reported that podoplanin knockout mice showed epicardial EMT and cardiac defects, including a perforated septum^70^. This evidence might be explained by the fact that the cells derived from the epicardium located at the atrioventricular junction populate atrioventricular cushions and form the atrioventricular sulcus, which is necessary for correct septation^71^.

The involvement of TFEB in the balance between the epithelial and mesenchymal phenotypes has already been described. In 3T3 cells and mouse embryonic fibroblasts, TFEB was required for E-cadherin promoter activation, and when overexpressed, TFEB upregulated WT1 and downregulated the EMT activator Snail^72^. In contrast, in gastric cancer, TFEB promoted EMT by Wnt/β-catenin signaling activation^73^.

However, our findings provide the mechanistic insights into how TFEB regulates EMT orchestrated by TGFβ. Indeed, in murine epicardial cells, overexpressed TFEB impaired TGFβ-driven EMT by positively regulating the expression of the *Tgif1* corepressor, as demonstrated by the rescue experiment (Fig. 6). Interestingly, the ChIP-seq analysis revealed that TFEB also binds to the promoters of two other SMAD transcriptional corepressors, *Ski* and *Skil*; however, we showed that these molecules are unlikely to mediate the inhibitory effect of TFEB on EMT in MECs (Supplementary Fig. 8). It is tempting to speculate that TFEB may activate different SMAD repressors depending on the cell context. This hypothesis is further supported by our findings that the regulatory role of TFEB in EMT has also been observed in vascular ECs and in epithelial MDCK cells (Fig. 9).

TGIF1 upregulation was previously shown to drastically inhibit TGFβ-induced α-SMA expression and fibrotic reactions^74,75^, similar to our observations. TGIF1 not only prevents target gene expression activation but also actively represses the promoters^45^. We also observed the downregulation of the mesenchymal signature in MECs overexpressing TFEB S142A and its upregulation in *Tfeb*-silenced cells even in the absence of an exogenous TGFβ stimulus (Fig. 4).

To understand the physiological role of TFEB in TGIF1 regulation of TGFβ signaling, we first investigated the dynamics of TGIF1 protein levels after TGFβ stimulation in MECs. TGFβ1 treatment caused a rapid proteasomal degradation of TGIF1, which is necessary to lift the transcriptional repression, followed by de novo mRNA synthesis, leading to the restoration of the protein level at 1 h and a further increase at 24 h, presumably to stop the TGFβ response. A similar pattern has already been demonstrated for *Ski* and *Skil*^56,58^. Then, we compared the effect of TFEB loss of function to that of TGIF1. TGIF1 is required to set the maximum ceiling to which the TGFβ signal can activate transcription; thus, TGIF1-silenced cells should produce more transcripts in response to a TGFβ stimulus^44^. However, it was also observed that TGIF1-silenced cells responded strongly to lower doses of TGFβ^44^. The same behavior was reported for another SMAD corepressor, SKIL. *SKIL*-silenced renal tubular epithelial cells responded to much lower concentrations of TGFβ than control cells^76^. In the *Tgif1*-silenced MECs, we observed both a dramatic decrease in the threshold responsiveness to TGFβ1 and the amplification of TGFβ1 signaling at higher doses. The pattern of the TGFβ1 dose-response was the same in the *Tfeb*-silenced cells but was weaker, which is understandable, as *Tfeb* silencing reduced but did not completely eliminate TGIF1 protein expression. Similar sensitization to TGFβ stimulus in terms of αSMA expression was observed in primary epicardial cells from *Gata5*^*+*^*; Tfeb* ^*flox/flox*^ mice. Altogether, we propose a model in which TFEB in epicardial cells determines the necessary quantity of TGIF1 protein required to set the right threshold for TGFβ signaling. TGFβ1-induced TFEB downregulation, observed at 48 h, does not have any impact on TGIF1 regulation of EMT onset, which occurs in the first hour of TGIF1 treatment, but rather is a consequence of the differentiation to mesenchymal cells where TFEB might no longer be needed.

While the involvement of TGFβ signaling in the activation of epicardial EMT was abundantly demonstrated in vitro^10,13,77^, its exact role in in vivo models remains unclear. The full single individual knockout of TGFβ ligands did not cause coronary vessel defects, but this finding may be attributed to their overlapping expression and functional redundancy^78^. More intriguing data were produced in models of deletion of other components of the TGFβ signaling pathway, suggesting its role in different aspects of EPDC biology. Deletion of the TGFβ receptor Alk5 in the epicardium under the Gata5 promoter resulted in a thin and poorly attached epicardial layer at E12 and reduced expression of N-cadherin^13^. At E15, mutant hearts lacked smooth muscle cell coverage of coronary vessels. As EMT intensity was not specifically analyzed in this study, it is difficult to determine whether TGFβ signaling is required for EMT or later differentiation into vSMCs. However, the primary cells from Alk5/Gata5-Cre mutant embryos failed to undergo TGFβ-induced EMT in vitro. Similarly, SMAD4 deletion in epicardial cells under the WT1 promoter resulted in a reduction in cardiac fibroblast number^79^, but as the analysis was performed at E18.5, it is not possible to distinguish between an EMT or a later differentiation defect. We found that the mice with *Tfeb* deletion in the epicardium were viable and morphologically normal, without alterations in the EMT process at E13.5, but with premature differentiation and myocardial invasion of vSMCs and fibroblast precursors, which, however, was recovered at later developmental stages. In contrast, in the *Gata5*^*+*^*; Tfeb*^*fs*^ embryos, we observed an inhibition of EMT at E13.5 and a reduced number of vSMCs and fibroblasts in the myocardium at E15.5. The latter effect might be a consequence of the inhibited EMT or a cumulative effect of the impairments of both EMT and subsequent cell invasion and differentiation. Thus, the phenotypes of *Tfeb*-deleted and *Tfeb*-overexpressing embryos suggest TFEB involvement in the regulation of both EMT and EPDC differentiation, which may be explained by the hypothesis that both are regulated by TGFβ signaling or, alternatively, that TFEB might also regulate other signaling pathways.

*Gata5*^*+*^*; Tfeb* ^*flox/flox*^; and *Gata5*^*+*^*; Tfeb*^*fs*^ mutants did not show a specular phenotype, so it is interesting to speculate about why EMT defects are absent in *Tfeb*-deleted animals. Compensation by TFE3 for the lack of TFEB was previously described^80^, but we can exclude this possibility, considering that the TFE3 expression level in epicardial cells was 2–4 times lower than that of TFEB (Fig. 2d). There might be other hypothetical explanations for the lack of a morphological phenotype in the *Gata5*^*+*^*; Tfeb* ^*flox/flox*^ mice. First, in vivo, the potential increased response of the cells to TGFβ is not translated into an increased EMT without an upstream TGFβ stimulus, which, although not fully elucidated, might be temporally and spatially regulated^81^. For instance, hypoxia^69^ and Notch signaling^24^ regulate autocrine TGFβ expression. Furthermore, the other TGFβ signaling repressors SKI and SKIL could compensate for the TGIF1 decrease. In addition, in vivo, only a minute population of epicardial cells undergoes EMT, suggesting the presence of other probably not yet identified restrictive mechanisms in most cells. Accordingly, *Tgif*^−/−^ mice were viable and did not present any morphological abnormalities^82,83^, except for a severe defect in placental vascularization, intriguingly similar to that observed in *Tfeb* null mice^38^. Finally, the few studies that report increased epicardial EMT in mouse models describe a mild phenotype^84,85^. For example, the epicardium-specific deletion of the Ras GTPase-activating protein Nf1 led to earlier and more robust EMT, resulting in hearts with amplified coverage of coronary capillaries; otherwise, the mutant mice were healthy and did not present any defects^84^.

Altogether, these data shed light on the mechanics of EMT regulation in the epicardium, indicating that *Tfeb* modulates cell sensitivity to TGFβ by upregulating the expression of *Tgif1*. The effect of TGFβ family signaling strongly depends on the cellular context^86^. This pleiotropic function exerted by a relatively simple signaling cascade is achieved by a variety of SMAD coactivators and corepressors that dictate the choice of target genes^87^. Therefore, the maintenance of balanced expression levels of these regulators in specific cell types and times is definitive for the signaling outcome. There are many examples showing that TGIF1 quantity variation fine-tunes TGFβ and retinoic acid signaling in specific cell contexts and has a profound impact on cell fate^83,88–90^.

Given the emerging view of TFEB as a therapeutic target due to its ability to activate autophagy and cell clearing, a possible role for TFEB in counterbalancing EMT, which is crucial not only in injured heart repair but also in tumor progression and tissue fibrosis, should be thoroughly investigated.

## Methods

Our research complies with all relevant ethical regulations: the protocol of isolation of primary human ECs was approved by the Office of the General Director and Ethics Committee of the Azienda Sanitaria Ospedaliera Ordine Mauriziano of Torino Hospital and the ethics committee of the University of Turin and the Italian Ministry of Health approved the animal study.

### Antibodies

Anti-GAPDH (6C5), anti-GFP (IF), anti-SM22α, and anti-TBX18 were purchased from Abcam. Anti-CD31 was purchased from BD Pharmingen. Anti-TFEB (WB) was purchased from Bethyl Laboratories. Anti-Cre Recombinase (D7L7L), anti-Flag tag, anti-PDGFRα (D1E1E), anti-PDGFRβ (28E1), anti-Slug (C19G7), anti-TFEB (ChIP-seq), and anti-Vimentin (D21H3) were purchased from Cell Signaling Technology. Anti-TGIF1 (H-172) and anti-WT1 (C19) were purchased from Santa Cruz Biotechnology. Anti-αSMA (1A4) and anti-vinculin (V9131) were purchased from Sigma-Aldrich. Anti-GFP (IHC), anti-cTnT, and anti-ZO1 were purchased from Thermo Fisher Scientific. HRP goat anti-mouse and goat anti-rabbit secondary antibodies (WB) were purchased from Jackson ImmunoResearch Laboratories. EnVision+ System- HRP Labelled Polymer Anti-Rabbit (IHC) was purchased from Dako. Alexa Fluor 555 donkey anti-mouse, Alexa Fluor 488 donkey anti-rabbit, Alexa Fluor 647 goat anti-rat and Alexa Fluor 488 goat anti-chicken secondary antibodies (IF) were purchased from Thermo Fisher Scientific. Catalog numbers, dilutions and validation information for the antibodies is reported in Supplementary Table 1.

### Reagents

Doxycycline and lactacystin were purchased from Sigma-Aldrich. Collagen I (rat tail) was purchased from Roche. Recombinant human TGFβ1, TGFβ2, and recombinant mouse TGFβ1 were obtained from RnD Systems. DAPI and TO-PRO-3 Iodide nuclear stains were purchased from Thermo Fisher Scientific.

### Mice

All animal procedures were approved by the ethics committee of the University of Turin and by the Italian Ministry of Health (protocol approval no. 864/2015‐PR). All animals were housed in individually ventilated cages supplied with enrichment. The facility ambient conditions were: temperature: 22 ± 2 °C, humidity: 55 ± 15%, light cycle 12 h:12 h, daylight started from 7.00 a.m. All mice were kept on C57BL/6 background. To generate embryos, females and males of appropriate genotypes aged between 9 and 18 weeks were mated. Embryos were staged according to the day of plug formation. Genotyping was performed on yolk sack tissue. Four wild-type mice (8 weeks old, both sexes) were used for the experiment in Fig. 1c; six females and three males were used to generate embryos for the experiments in Fig. 1b–e, Fig. 2d, and MECs derivation. The generation of *Tfeb*^*EGFP*^ mice was described in ref. 33. Homozygous mice were used in the experiments. 4 adult *Tfeb*^*EGFP*^ mice (8 weeks old, both sexes) were used for the experiment in Fig. 1c; five females and three males were used to generate embryos for the experiments in Fig. 1b–e. Epicardium-specific *Tfeb*-overexpressing *Gata5*^*+*^*; Tfeb*^*fs*^ mice were obtained by crossing *Tfeb-flag*^*fs*^ mice^48^ with *Gata5-Cre* mice^8^, which were a kind gift from P. Ruiz-Lozano (Stanford University, USA). Heterozygous animals of both genotypes were mated to generate embryos. *Gata5*^*+*^ littermates were considered controls. 40 females and 20 males were used to generate embryos for the experiments in Table 1 and Figs. 2b–f, 3a, b, and 6d, e. Epicardium-specific deletion was achieved by crossing *Gata5-Cre* mice with *Tfeb* ^*flox/flo*x^ mice ^91^ and backcrossing the resultant *Gata5*^*+*^*; Tfeb* ^*flox/+*^ offspring with *Tfeb* ^*flox/flo*x^ mice. *Tfeb* ^*flox/flo*x^ littermates were considered controls. 24 females (12 *Gata5*^*+*^; *Tfeb* ^*flox/+*^; and 12 *Tfeb* ^*flox/flo*x^) and 12 males (6 *Gata5*^*+*^; *Tfeb* ^*flox/+*^; and 6 *Tfeb* ^*flox/flo*x^) were used to generate embryos for the experiments in Fig. 8c–e.

### Primary epicardial cell culture

Primary epicardial cells were isolated as previously described^11^. E11.5 hearts were dissected, atria and great vessels were removed, and ventricles were placed on collagen-coated dishes or glass coverslips in DMEM, 1% penicillin/streptomycin, and 10% FBS. After 24 h, the hearts were removed, and attached epicardial cells were cultured in DMEM with 10% FBS for the indicated times. For EMT induction, epicardial monolayers were starved overnight in DMEM and then treated with the indicated concentration of TGFβ1 in DMEM containing 10% FBS for 48 h.

### Derivation of the MEC line from primary epicardial cells

For the derivation of the MEC line, we followed the method described in^47^. Briefly, ventricular tissue of several E13.5 hearts was dissected, placed on gelatin-covered dishes and cultured in DMEM, 1% penicillin/streptomycin, and 15% FBS. After 4 days, heart tissue was removed, and attached epicardial monolayers were cultured until confluence. Then, the cells were replated several times, and colonies with an epithelial morphology were manually picked. The resultant MEC line was cultured in DMEM with 10% FBS and maintained its morphology after many passages. For induction of EMT, MECs were seeded at 200,000 cells in a six-well plate or 40,000 cells on a gelatin-coated coverslip, starved overnight in DMEM containing 2% FBS, and then stimulated with the indicated concentration of TGFβ1 in DMEM containing 2% FBS for the indicated time periods.

### Human endothelial cell culture

Human endothelial cells (ECs) were isolated from umbilical cord veins as previously described^92^. Briefly, umbilical vein was cannulated with a blunt 17-gauge needle and the needle was secured by clamping the cord over the needle. The vein was perfused with 50 ml of PBS to wash out the blood. A total of 10 ml of 0.2% collagenase A (Roche Diagnostics) in cell culture medium was then infused into the vein and incubated 30 min at room temperature. The collagenase solution containing the ECs was flushed from the cord by perfusion with 40 ml of PBS, collected in a sterile 50 ml centrifuge tube and centrifuged 5 min at 800 × *g*. ECs were grown in M199 medium supplemented with 20% FCS, EC growth factor (100 g/ml; Sigma-Aldrich), and porcine heparin (100 g/ml; Sigma-Aldrich). Pools of five different donors were used to minimize cell variability. The isolation of primary human ECs was approved by the Office of the General Director and Ethics Committee of the Azienda Sanitaria Ospedaliera Ordine Mauriziano of Torino Hospital (protocol approval no. 586, 22 October 2012; no. 26884, 28 August 2014; and no. 1494 del 9 July 2018), and informed consent was obtained from each patient. For the EndMT induction experiment, ECs were seeded in gelatin-coated 6-multiwell plates, with 100,000 cells per well, starved overnight in M199 with 2% FCS, and treated with 20 ng/ml TGFβ1 or TGFβ2 in M199 with 5% FCS for the indicated periods of time.

### MDCK culture

The Madin Darby canine kidney cell line (MDCK) was purchased from ATCC (CCL-34) and was maintained in DMEM with 10% FBS. For induction of EMT, 100,000 cells were seeded in a six-well plate, starved overnight in DMEM containing 2% FCS, and treated with 10 ng/ml human TGFβ1 in DMEM containing 2% FCS for the indicated periods of time.

### Genetic manipulation and biological assays

The *TFEBS142A* mutant was generated from TFEB cDNA (Origene, cod. SC122773) by inserting a single point mutation using the Phusion Site‐Directed Mutagenesis Kit. TFEBS142A was cloned into the pTRIPZ inducible vector, and lentivirus particles were produced according to ref. 93. Cells were infected with lentivirus at an MOI of 1 and selected with 2 µg/ml puromycin. The transgene was induced by doxycycline addition (0.5 μg/ml) for 8 h for ChIP‐seq experiments or 24 h prior to TGFβ1 treatment for EMT induction experiments. Cells infected with pTRIPZ‐TFEBS142A but not treated with doxycycline were used as a control and are indicated as the “control”.

Loss‐of‐function experiments were carried out with shRNA against *Tfeb* (TRCN0000013110) (for TGFβ1 dose-dependent experiments, see Figs. 8a and S6f) and *Tgif1* (TRCN0000055048, TRCN0000218560, TRCN0000233980) cloned into the pLKO.1‐puro vector (Sigma-Aldrich). Lentiviral particle production and cell infection were performed as described above. Cells infected with pLKO.1‐puro nontargeting RNA vector were used as a control. *Tfeb* silencing in other experiments was performed by transfecting siRNAs (SASI_Mm02_00320900, SASI_Mm01_00082195, SASI_Mm01_00082196 from Sigma-Aldrich) with Lipofectamine RNAiMAX (Thermo Fisher Scientific) following the manufacturer’s instructions. Cells transfected with nontargeting siRNA (SIC001, Sigma-Aldrich) were used as a control. *Skil* was silenced by transfection of specific siRNA (EMU063011, Sigma-Aldrich) with Lipofectamine RNAiMAX (Thermo Fisher Scientific) following the manufacturer’s instructions. Cells transfected with GFP-targeting siRNA (EHUEGFP, Sigma-Aldrich) were used as controls. In all cases, infection or transfection of the cells was performed 48 h prior to sample collection or TGFβ1 treatment in EMT experiments.

The cell proliferation rate was evaluated by a BrdU assay (Cell Signaling Technology) according to the manufacturer’s protocol. MEC TFEB S142A cells were seeded in 96-well plates at a density of 2000 cells/well, TFEBS142A expression was induced with doxycycline for 24 or 48 h, and the cells were incubated with BrdU for 24 h before detection.

### Luciferase assay

The mouse *Tgif1* promoter sequence (pTGIF1-full) and the promoter with a deleted TFEB-binding region (pTGIF1-del) were cloned into the luciferase-expressing vector pGL4-luc2P-Hygro (Promega). The *Tgif1* promoter was defined as a sequence (−1106; +310) from the TSS of *Tgif1* transcript variant 1. The TFEB binding peak according to ChIP-seq data is situated at −438 and +187. The central 321 bp of the peak containing a possible TFEB-binding motif agcatgtgag according to the Jaspar algorithm (score 9.3) was deleted in the pTGIF1-del construct. pTGIF1-full and pTGIF1-del were electroporated into MEC-TFEBS142A cells with an Amaxa electroporator (Lonza), and the cells were selected with 0.5 mg/ml hygromycin. Cells were seeded in 96-well plates (5000 cells per well) and stimulated with doxycycline for the indicated time intervals, and luciferase activity was analyzed with a Luciferase Assay System Kit (Promega) using a Glomax 20/20 luminometer (Turner Biosystems, Sunnyvale, CA, USA). The relative reporter activity was calculated by normalizing the luciferase activity in doxycycline-treated cells to that in untreated cells.

### Tissue and cell staining and analysis

For immunohistochemistry, embryos were fixed in 4% paraformaldehyde overnight, washed with PBS, dehydrated, embedded in paraffin and cut into 10 µm sections. Sections were rehydrated and processed for heat-induced antigen retrieval in R-Universal buffer (BioVendor) in a 2100 Antigen Retriever (BioVendor). Then, the sections were permeabilized and saturated in PBS with 0.2% Triton X‐100 and 5% goat serum for 1 h at room temperature (RT), quenched with 3% H_2_O_2_ for 10 min at RT, immunostained with primary antibody diluted in PBS with 0.2% Triton X‐100 and 5% BSA overnight at 4 °C, washed, incubated with secondary antibody for 1 h at RT, washed, developed with DAB solution (DAKO) and mounted.

For immunofluorescence, embryos were fixed for 24 h in zinc fixative (0.5% zinc chloride, 0.5% zinc acetate, 0.05% calcium acetate in 0.1 M Tris, pH 7.4), dehydrated in 30% sucrose overnight, frozen in OCT compound and cut into 10‐μm thick sections. Sections were permeabilized and saturated in PBS containing 0.2% Triton X‐100 and 5% donkey serum for 1 h at RT, immunostained with primary antibody diluted in PBS containing 0.2% Triton X‐100 and 5% BSA overnight at 4 °C, washed, incubated with secondary antibody for 1 h at RT, washed, and mounted.

Cells were grown on appropriately coated coverslips, washed with PBS with Ca2^+^ and Mg2^+^, fixed in 4% paraformaldehyde for 10%, washed, permeabilized in PBS with 0.1% Triton X‐100 for 2 min on ice, washed, incubated with primary antibody diluted in PBS with 5% BSA and 5% donkey serum for 1 h at RT, washed, incubated with secondary antibody for 1 h at RT, washed, and mounted.

Immunofluorescence images were acquired on TCS SPE or TCS SP8 confocal laser‐scanning microscopes (LAS AF software, Leica Microsystems). Different fields per sample section were randomly chosen for analysis. When the same molecule was evaluated in different samples, laser power, gain, and offset settings were maintained. Images were quantified using ImageJ software. The mean immunofluorescence intensity in whole cells or in nuclei was obtained by selecting the cell area (threshold on the quantified channel set to select all cell areas) or nuclear area (threshold on the nucleus stain set to select the nuclei area). The cell surface was quantified by normalizing the area occupied by cells in an image to the number of nuclei. The depth of invasion of PDGFRα^+^ and PDGFRβ^+^ cells in the E15.5 embryos (Fig. 3e) was quantified by manually measuring the shortest distance from the surface for each cell. The number of invaded PDGFRα^+^ and PDGFRβ^+^ cells (Fig. 3e) was measured by manually counting the number of positive cells in an image normalized to the length of the heart surface.

### ChIP-seq analysis

Chromatin immunoprecipitation of TFEB was performed as previously described^33^. Approximately 2 × 10^7^ crosslinked cells were resuspended in 250 μl of SDS lysis buffer (10 mM EDTA, 1% SDS, 16.7 mM Tris pH 8) with protease inhibitors and incubated for 10 min on ice. After sonication, the cell lysate was centrifuged at 12,000 *g* for 10 min at 4 °C. The supernatant was diluted tenfold with ChIP dilution buffer (16.7 mM Tris–HCl, pH 8.0, 167 mM NaCl, 1.2 mM EDTA, 1% Triton) before immunoprecipitation. The supernatant was incubated with 5 μg of anti‐TFEB antibody or IgG with rotation at 4 °C for 16 h. Samples treated with IgG were used as a negative control. Afterward, previously BSA‐saturated beads (Dynabeads® Protein G) were added for 2 h. Immunoprecipitated complexes were extensively washed before adding SDS elution buffer (50 mM Tris–HCl, pH 8.0, 10 mM EDTA, 1% SDS, 5 mM DTT, 150 mM NaCl) for 30 min at room temperature. After decrosslinking, DNA was purified using a QIAQuick PCR Purification Kit according to the manufacturer’s instructions.

For genome‐wide analysis of TFEB binding, sequencing libraries were constructed using the NEBNext® ChIP‐seq Library Prep Reagent Set for Illumina and an Illumina HiScanSQ sequencer. ChIP‐seq reads were aligned to the mm9 genome assembly using Bowtie v0.12.7 with the following parameters: ‐q –max/dev/null ‐v 1 ‐S –sam‐nohead ‐m 1. Data were filtered using the following specifications. Duplicate reads were filtered out. BedGraph files were generated by using the MACS tool. Peak calling was performed as described previously^94^ using a p value cutoff = 1E‐05. HOMER and GREAT software were used for ChIP-seq peak annotation and analysis, while the Jaspar database was used as a source for the localization of TFEB binding sites. For the annotation of TFEB peaks on promoter regions of mouse genes, GREAT was used with the following parameters: association rule basal+extension: 2500 bp upstream, 2500 bp downstream, 0 bp max extension. Motif enrichment was performed with HOMER using the -size given -len 8 options. The Jaspar MA0692.1 matrix was used to identify TFEB binding sites on DNA sequences.

### Micro-CT

Micro-CT analysis was performed on E15.5 mouse embryos using a Bruker Skyscan 1172 micro-CT. Embryos were fixed in formalin and then stained for 15 days with a soft tissue contrast agent [phosphotungstic acid (PTA) 2.5% dissolved in water]. Acquisitions were performed at 80 KV using a 0.5 mm Al filter at a resolution of 7 µm, 0.6° rotation step, 360° scan, and 4x frame averaging. Three-dimensional reconstruction was performed by using NRecon software; images were analyzed with DataViewer and CTvox (Bruker). Eight *Gata5*^*+*^*; Tfeb*^*fs*^ and 8 control embryos were analyzed. The thickness of the compact myocardium of the right and left ventricles and interventricular septum was measured in the same transversal plane at the level of the atrioventricular valves. Pericardial cavity size was evaluated in the same frontal plane and reported as the area of pericardial cavity not occupied by the heart appearing in the image.

### Western blot analysis

Cells were washed twice with PBS and lysed in 10 mM Tris-HCl (pH 8.0) with 1% SDS buffer heated to 95 °C. Lysates were sonicated for 10 min (Branson SLP), and protein concentration was measured by Pierce BCA assay (Thermo Fisher Scientific). A total of 10–30 µg of lysate was resolved by SDS-PAGE and transferred to PVDF membranes with a Trans Blot Turbo System (Bio-Rad). The membranes were dried at RT, rehydrated in TBS 0.1% Tween, immunostained with primary antibody overnight at 4 °C, washed, incubated with secondary antibody for 1 h at RT, washed and developed with ECL substrate (Bio-Rad). Images were acquired with a ChemiDoc Touch Imaging System (Bio‐Rad) and analyzed with Image Lab software 5.2.1 (Bio‐Rad). At least three independent replicates for an experiment were used. Uncropped membrane scans are provided in Source data.

### Quantitative PCR

The cells were washed twice with PBS, and RNA was extracted with a Maxwell RSC System (Promega). Epicardial explants of the same genotype were pooled together. The RNA concentration was measured with a Nanodrop ND‐100 (Nanodrop Technologies) and retrotranscribed with a High Capacity cDNA Reverse Transcription kit (Thermo Fisher Scientific). Real-time PCR was performed with TaqMan Gene Expression Master Mix (Thermo Fisher Scientific) or PowerUp Sybr Green Master Mix (Thermo Fisher Scientific) depending on the gene analyzed using CFX96 (Bio‐Rad) and 7500 Real-time PCR system (Thermo Fisher Scientific). GAPDH or TBP was used as an internal control to normalize gene expression levels. The sequences or purchase codes of the probes used are listed below.

Acta2 (5′- GAGAAGCCCAGCCAGTCG-3′, 5′- CCAGTTGGTGATGATGCCGT-3′), Actb, Adamts6 (qMmuCID0012947, Bio-Rad), Col16a1 (qMmuCID0024725, Bio-Rad), Col1a1 (qMmuCID0021007, Bio-Rad), Col5a3 (qMmuCID0024070, Bio-Rad), Col5a3 (qMmuCID0024070, Bio-Rad), Ctgf (5′- CCCTAGCTGCCTACCGACT-3′, 5′- GCCCATCCCACAGGTCTTAG-3′), Dcn (qMmuCID0039628, Bio-Rad), Itga11 (qMmuCID0021160, Bio-Rad), Gapdh, Itga5 (qMmuCID0015586, Bio-Rad), Ltbp2 (qMmuCID0023934, Bio-Rad), Mitf (Mm00434954_m1, Thermo Fisher), Mmp10 (5′-GCCCAGCTAACTTCCACCTT-3′, 5′-GATCCCCTTTGGGTAGCCTG-3′), Mmp15 (qMmuCID0026209, Bio-Rad), Mmp16 (qMmuCID0005967, Bio-Rad), Mmp2 (qMmuCID0021124, Bio-Rad), Mmp3 (5′-ATGGGCCTGGAACAGTCTTG-3′, 5′-AGTCCTGAGAGATTTGCGCC-3′), Pdgfrb (qMmuCID0025167, Bio-Rad), Pdpn (qMmuCID0011965, Bio-Rad), Plau (qMmuCID0022420, Bio-Rad), Ski (5′- CGCCGCACAAGTTCGTTG-3′, 5′- TTTTGGGTCTTATGGAAGCTGGG-3′), Skil (5′- TTTATGTTCAGCCCGACGCT-3′, 5′- TCCCGATGGTGTATCTGTCTTT-3′), Sparc (qMmuCID0023536, Bio-Rad), Tagln (5′- CTTCCAGCCCACAAACGACC-3’, 5′- AACTTGCTCAGAATCACGCCA-3′), Tbp (Mm01277042_m1, Thermo Fisher), Tgif1 (5′- GCCCCAAAAGAGAAACCAGTG-3′, 5′- AACAAGACCTTCCAGCTCCACA-3′), Timp2 (qMmuCID0017671, Bio-Rad), Tfeb (5′- TGCCCTGCCGACCTGACTCA-3′, 5′- TTCCAGCGCACGTCCAGGTC-3’), Tfec (Mm01161234_m1, Thermo Fisher), Tfe3 (Mm00552681_m1, Thermo Fisher), Tnc (qMmuCID0005706, Bio-Rad), Vcan (qMmuCID0005235, Bio-Rad), and Ywhaz (qMmuCED0027504, Bio-Rad).

### Data analysis

Statistical tests were performed, and plots were generated using GraphPad Prism software.

### Reporting summary

Further information on research design is available in the Nature Research Reporting Summary linked to this article.

## Supplementary information

Supplementary Information

Reporting Summary

## Data Availability

The ChIPseq dataset is available in The Gene Expression Omnibus of the National Center for Biotechnology Information (accession number GSE178575). The databases used in this study are available online: Jaspar [https://jaspar.genereg.net/], GREAT [http://great.stanford.edu/public/html/], HOMER [http://homer.ucsd.edu/homer/]. Source data for graphs and plots are provided as Source data file with this paper. Source data are provided with this paper.

## Source data

Source Data
