# Supplementary material for: The TFEB-TGIF1 axis regulates EMT in mouse epicardial cells
Source: Nat Commun. 2022 Sep 3;13:5191. doi: 10.1038/s41467-022-32855-3 (PMC9440911; doi:10.1038/s41467-022-32855-3)
Supplement: Supplementary file 1 — Supplementary Information [file 41467_2022_32855_MOESM1_ESM.pdf]

Supplementary information

The TFEB - TGIF1 axis regulates EMT in mouse epicardial cells
Astanina et al., 2022

Supplementary Figures

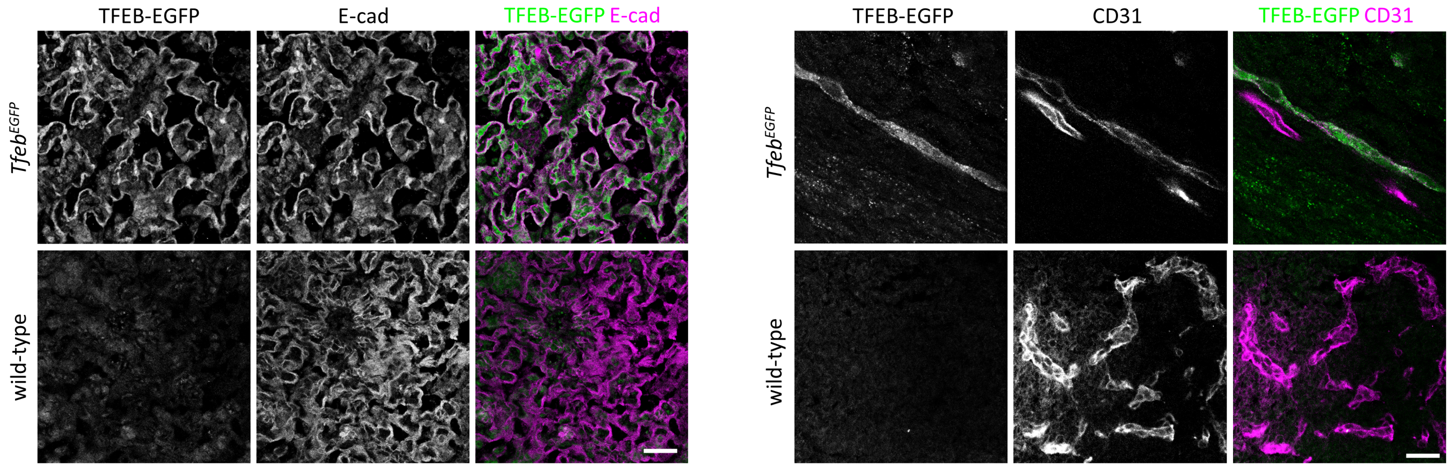

Supplementary Figure 1. ***Tfeb* is expressed in murine placental trophoblasts and endothelial cells**

Tfeb was expressed in E11.5 placental trophoblasts (left panel) and capillary endothelial cells (right panel). Immunofluorescence images of E11.5 wild-type and *Tfeb*^{EGFP} embryos stained for EGFP and E-cadherin or CD31. Four embryos for genotype were analyzed. The scale bar is 25 µm.

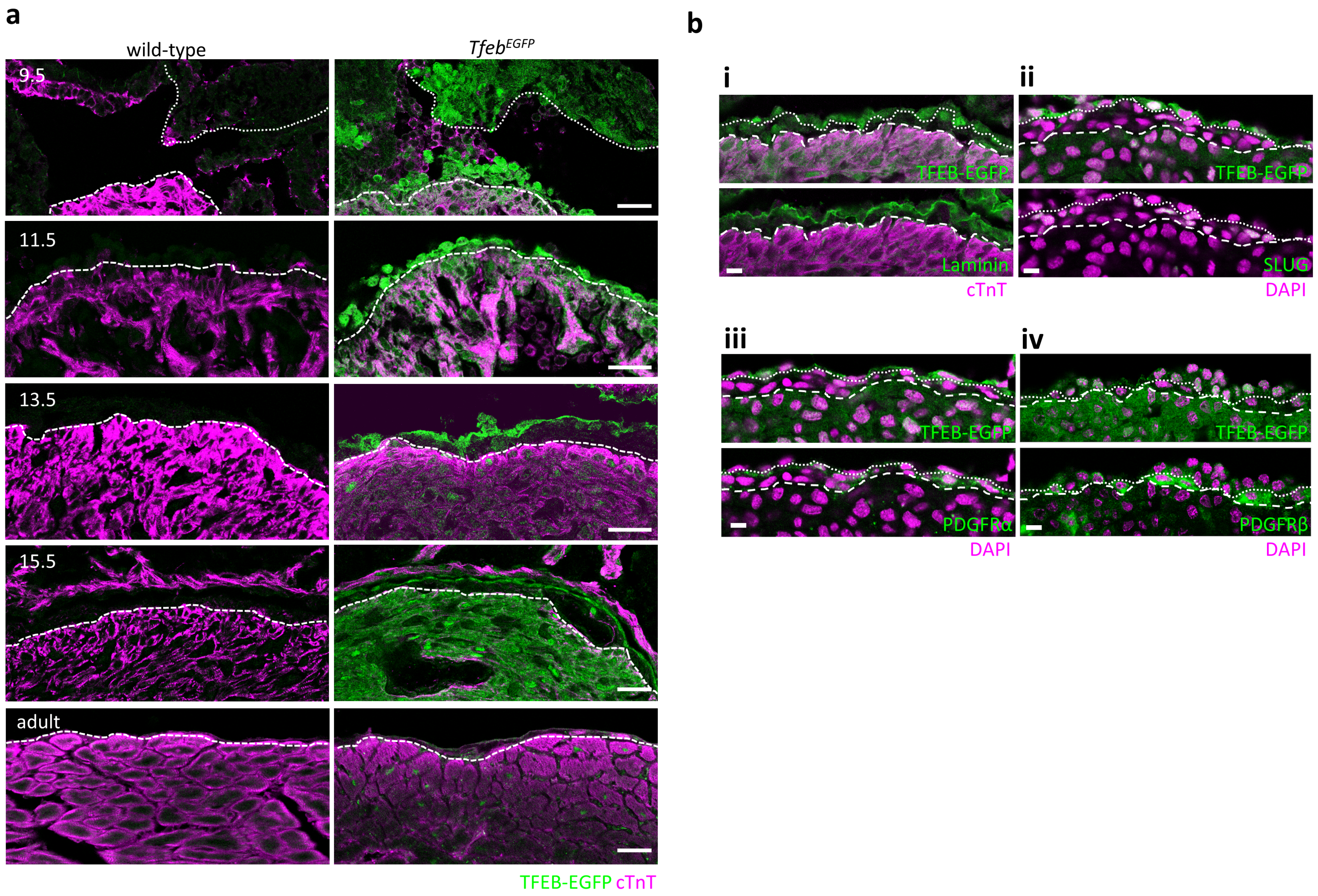

Supplementary Figure 2. *Tfeb* is expressed in murine epicardium and is downregulated in epicardial cells undergoing EMT (Supplementary images for Fig. 1)

a Immunostaining for EGFP (green) and cardiac troponin T (cTnT, magenta) in the hearts of wild-type and $Tfeb^{EGFP}$ mouse embryos at the indicated embryonic day and in adult mice. The dashed line surrounds the proepicardium at E9.5. Four embryos for genotype were analyzed. The scale bar is 25 µm.

b Immunostainings for EGFP, laminin, PDGFRα, PDGFRβ, SLUG (green) and DAPI (magenta) in the hearts of E13.5 $Tfeb^{EGFP}$ embryos. The dashed line indicates the myocardium surface, and the dotted line indicates the epicardium-subepicardium border. The scale bar is 25 µm.

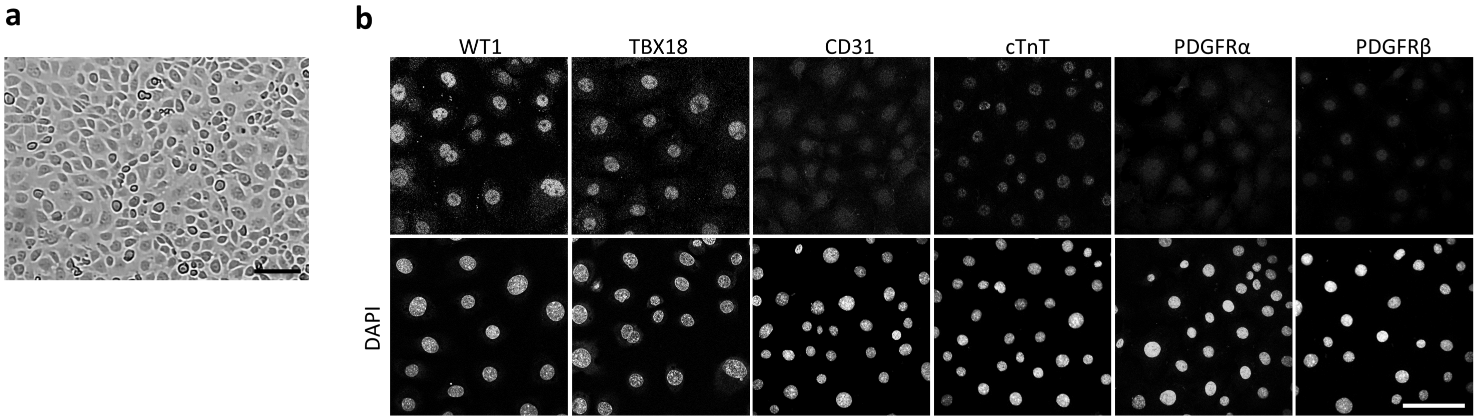

Supplementary Figure 3. MECs express epicardial markers

a A bright field microscopic representative image shows a typical cobblestone morphology of MECs. Scalebar is 200 μm.

b MECs express epicardial markers. MEC immunostaining for lineage markers. MECs were positive for epicardial markers (WT1 and TBX18) and negative for endothelial (CD31), cardiomyocyte (cTnT), fibroblast (PDGFRα) and SMC (PDGFRβ) markers. The experiment was repeated three times with similar results. The scale bar is 100 μm.

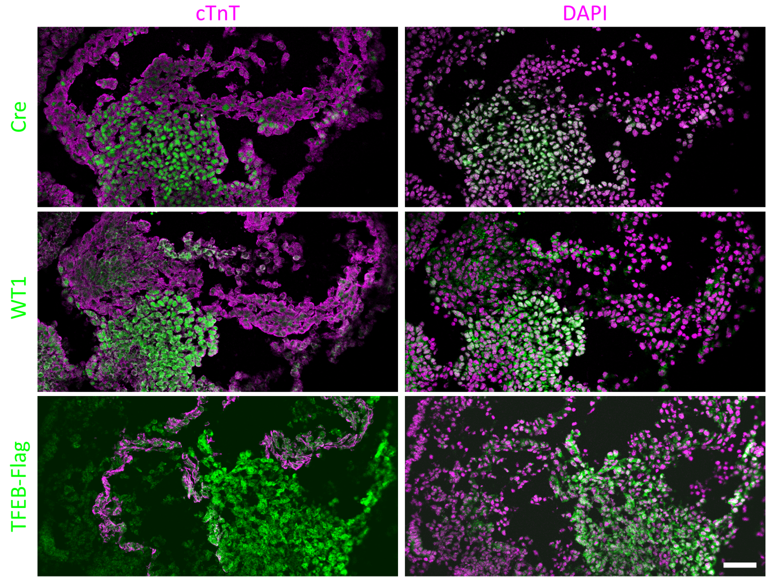

Supplementary Figure 4. The proepicardium in *Gata5⁺; Tfeb^fs* embryos at E9.5 expressed Cre recombinase and TFEB-Flag.

Immunofluorescence analysis for Cre, WT1 and TFEB-Flag proteins on consecutive sections of E9.5 *Gata5*$^{+}$*; Tfeb*^{fs} embryos. CTnT immunostaining and nuclei (DAPI) are shown in magenta. Four embryos were analyzed. The scale bar is 50 µm.

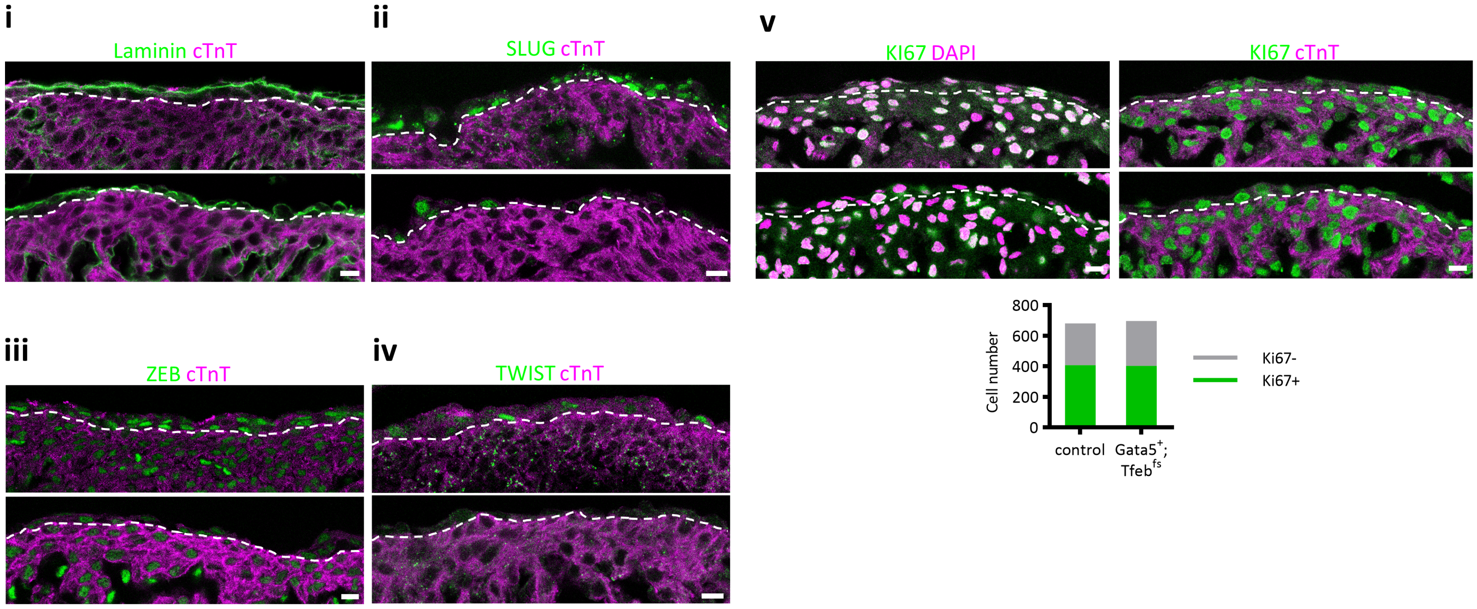

Supplementary Figure 5. *Tfeb* overexpression in the epicardium inhibits EMT at E13.5. (Supplementary images for Fig. 2)

E13.5 *Gata5*$^{+}$*; Tfeb*^{fs} embryos demonstrated inhibited epicardial cell delamination (**i**) and a reduction in the expression of the key EMT TFs Slug (**ii**), Zeb (**iii**), Twist (**iv**). No difference in epicardial cell proliferation (Ki67) was observed (**v**). **i-iv** Immunofluorescence analysis for laminin, Slug, Zeb, Twist (green) and cTnT (magenta) in E13.5 *Gata5*$^{+}$*; Tfeb*^{fs} and control embryos. The dashed line shows the myocardium surface. The scale bar is 10 µm. **v** Immunofluorescence analysis and quantification of proliferating Ki67-positive epicardial cells and EPDCs. Statistical significance was calculated with two-tailed Fisher's exact test, no significant difference was found.

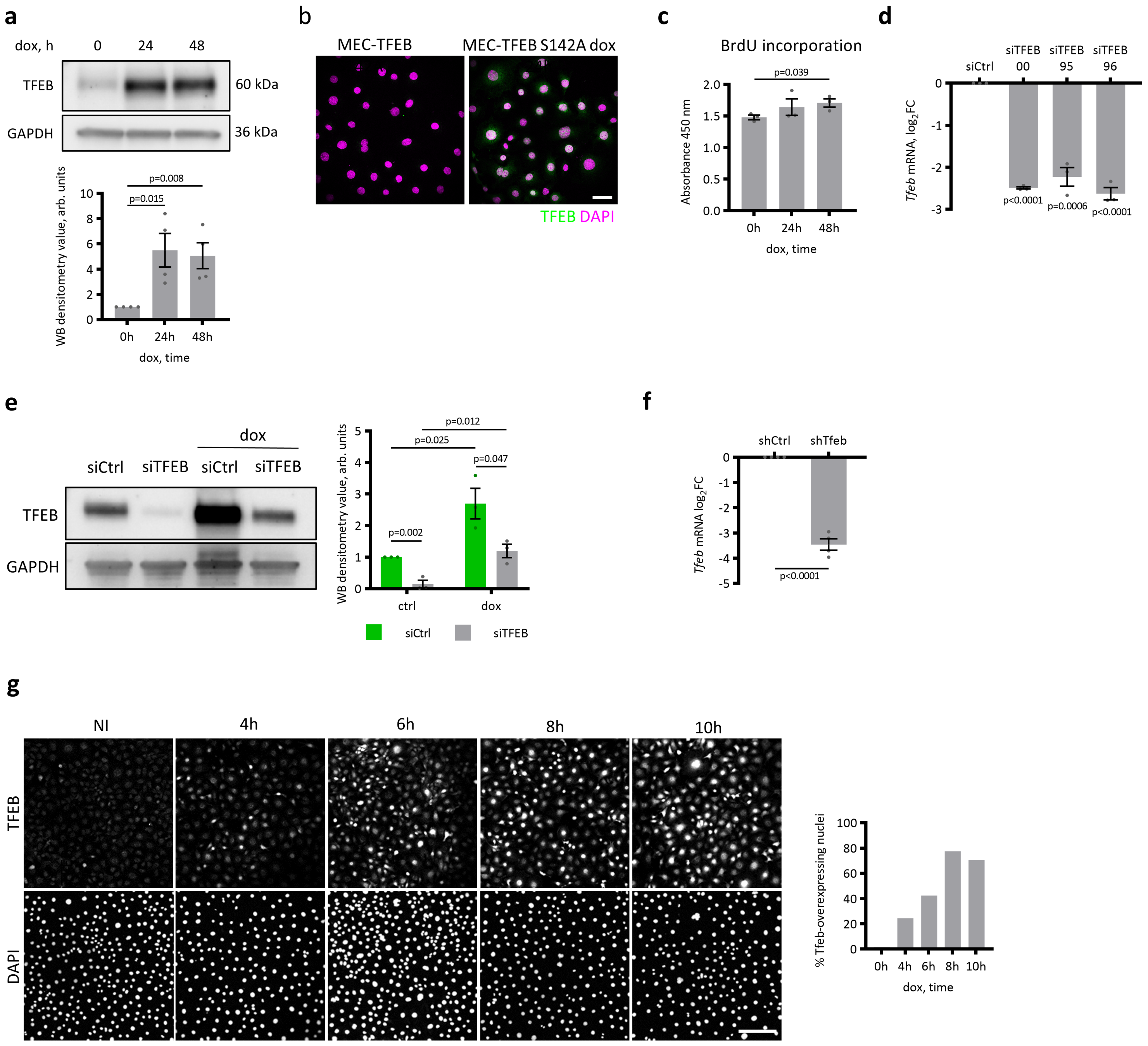

a
dox, h
0
24
48
TFEB
60 kDa
GAPDH
36 kDa
WB densitometry value, arb. units
p=0.008
p=0.015
0h
24h
48h
dox, time
b
MEC-TFEB
MEC-TFEB S142A dox
TFEB DAPI
c
BrdU incorporation
Absorbance 450 nm
p=0.039
0h
24h
48h
dox, time
d
siCtrl
siTFEB 00
siTFEB 95
siTFEB 96
Tfeb mRNA, log2FC
p<0.0001
p=0.0006
p<0.0001
e
dox
siCtrl
siTFEB
siCtrl
siTFEB
TFEB
GAPDH
WB densitometry value, arb. units
p=0.012
p=0.025
p=0.047
p=0.002
ctrl
dox
siCtrl
siTFEB
f
shCtrl
shTfeb
Tfeb mRNA log2FC
p<0.0001
g
NI
4h
6h
8h
10h
TFEB
DAPI
% Tfeb-overexpressing nuclei
0h
4h
6h
8h
10h
dox, time

Supplementary Figure 6. Characterization of TFEB S142A overexpression and *Tfeb* silencing in MECs

a Top, Western blot analysis of TFEB in MEC-TFEB S142A cells induced by doxycycline (dox) for 24 and 48 h. Bottom, densitometric analysis of Western blots; values are shown as the mean ±SEM, n=4. Student's two-tailed t test p values are reported in the plot.

b TFEB S142A is localized in the nuclei of MEC-S142A cells induced by doxycycline for 24 h. Immunostaining for TFEB (green); nuclei are stained with DAPI (magenta). Scale bar is 50 µm. The experiment was repeated three times with similar results.

c TFEB S142A overexpression has a negligible effect on cell proliferation, as measured by BrdU assays of MEC-S142A cells induced by doxycycline for 24 and 48 h. Values are shown as the mean ±SEM, n=3. Student's two-tailed t test p value is reported in the plot.

d Three siRNA sequences for *Tfeb* reduced *Tfeb* mRNA expression, as shown by real-time PCR of MECs 48 h after transfection with siRNAs. Values are shown as the mean ±SEM, n=3. Student's two-tailed t test p values are reported in the plot.

e Top, Western blot analysis of TFEB protein in MEC-TFEB S142A cells in which *Tfeb* was silenced and TFEB S142A was reintroduced by doxycycline induction (dox). Bottom, densitometric analysis of Western blots; values are shown as the mean ±SEM, n=3. Student's two-tailed t test p values are reported in the plot.

f *Tfeb* mRNA in MECs infected with a lentivirus coding for shRNA for *Tfeb* versus control shRNA, real-time PCR analysis. Values are shown as the mean ±SEM, n=4. Student's two-tailed t test p value is reported in the plot.

g Definition of the minimum time of doxycycline induction to obtain TFEB S142A overexpression in the majority of MECs by immunofluorescence analysis. MEC-TFEB S142A cells were induced with doxycycline (dox) for the indicated times and immunostained with an anti-TFEB antibody (left). The scale bar is 100 µm. The quantification of the percentage of TFEB S142A-overexpressing nuclei was performed with ImageJ software (right). The experiment was repeated two times with similar results.

Source data are provided as a Source Data file.

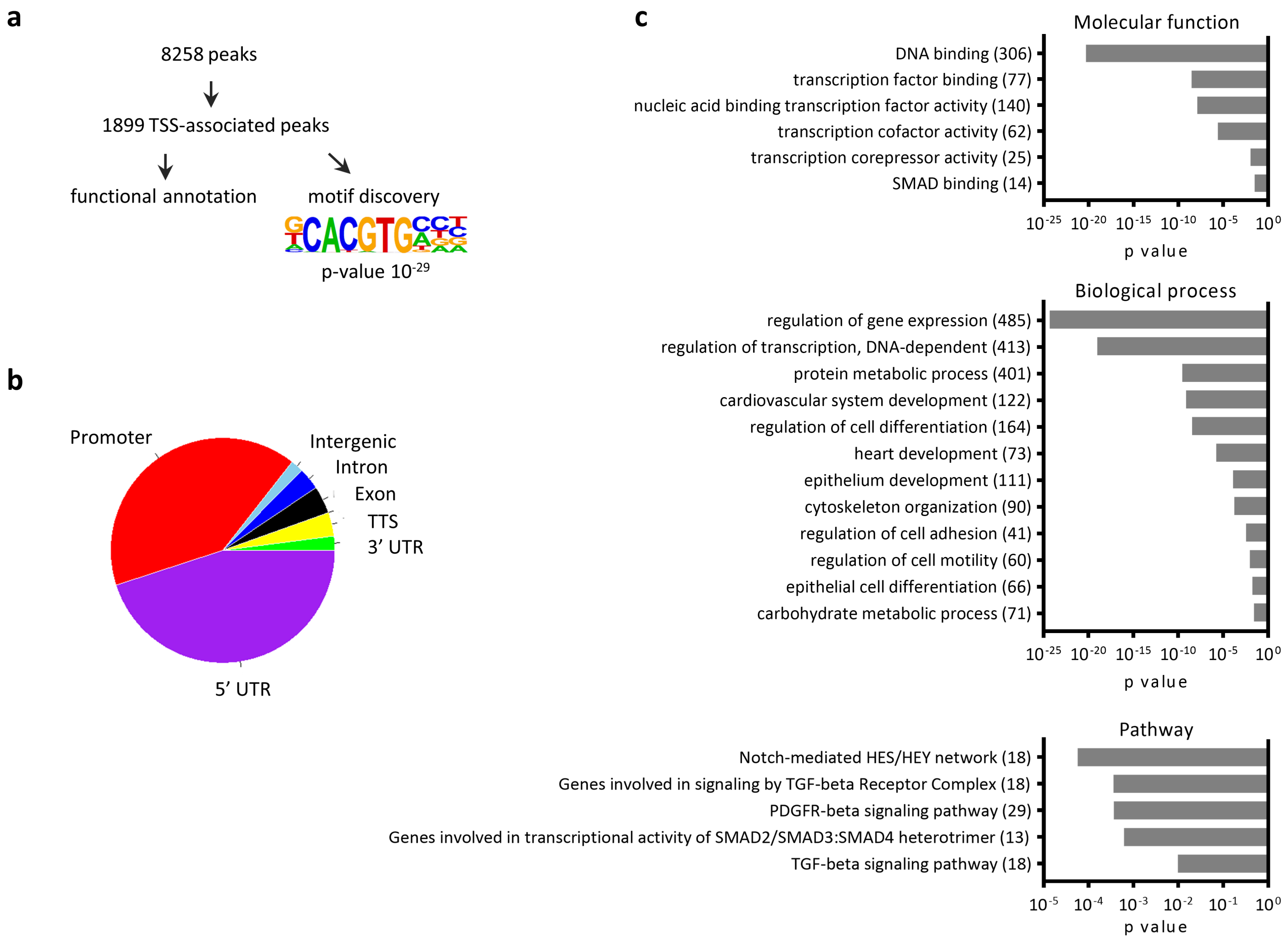

Supplementary Figure 7. ChIP-seq analysis of TFEB S142A-overexpressing MEC-TFEB S142A cells

a A pipeline of ChIP-seq data analysis. A total of 8258 peaks of TFEB S142A DNA binding were called, 1899 of which were superimposed mouse promoter regions (-2500 bp; + 2500 bp from TSS). TSS-associated peaks were selected for functional analysis and motif discovery analysis. Enriched E-box TFEB-binding motif matrix found in the sequences of the TSS-associated peaks by de novo motif discovery with HOMER software, p value 10^{-29}.

b Analysis of the enrichment of TFEB ChIP-seq peak distribution in the genome by HOMER software.

c Selected enriched GO categories correlated with the genes on the TSS region of which TFEB peaks were found by GREAT functional annotation analysis. Bars indicate FDR-corrected p values of the hypergeometric test, performed by GREAT software, numbers in parenthesis – gene number for each group.

Source data are provided as a Source Data file.

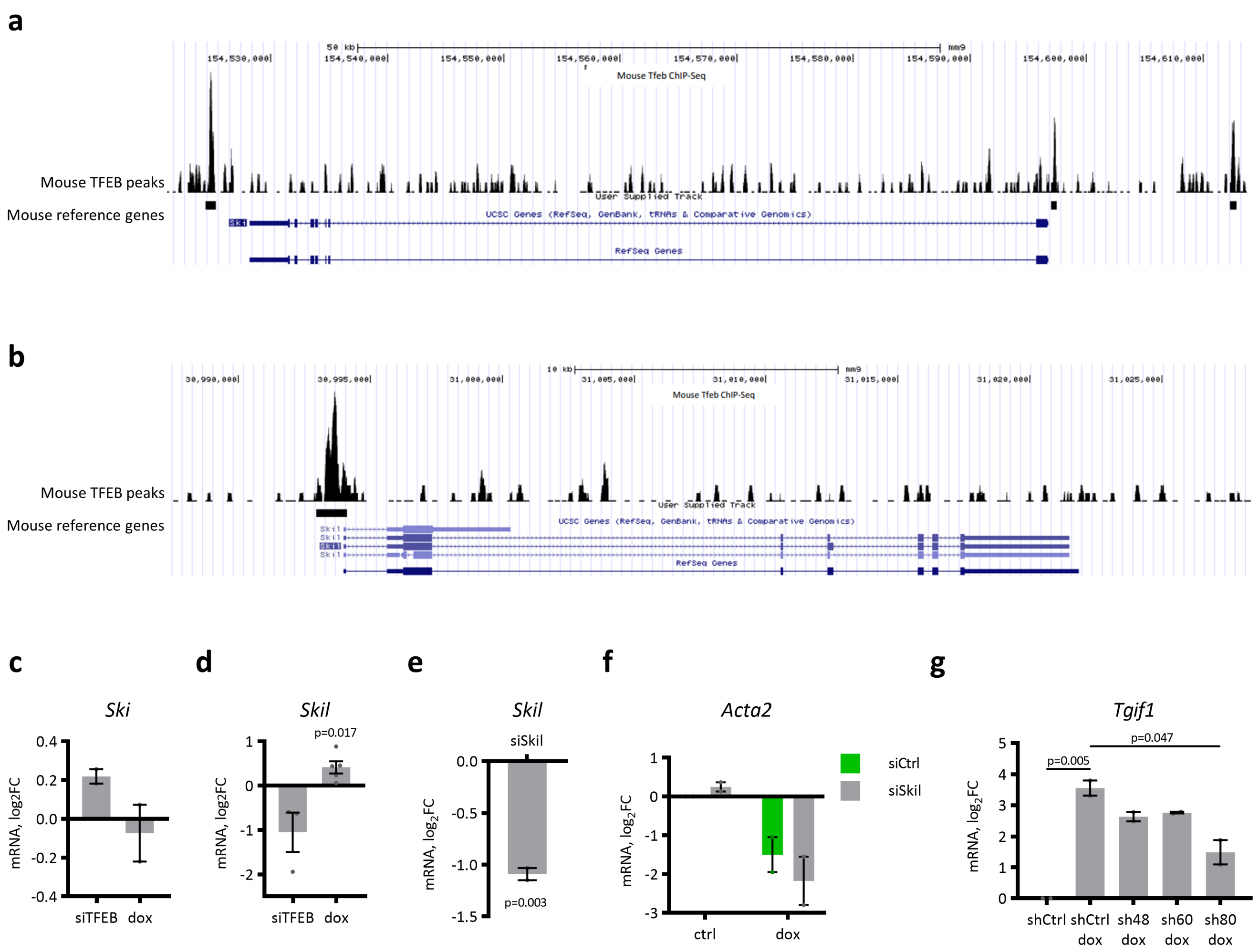

Supplementary Figure 8. ***Ski* and *Skil* do not mediate the TFEB S142A inhibitory effect on TGFβ1-induced *Acta2* transcription**

a-b Visualization of the peaks of TFEB binding in the region of the promoters of murine *Ski* (**a**) and *Skil* (**b**) genes in the UCSC genome browser.

c-f Validation of the *Ski* and *Skil* genes as possible mediators of the TFEB inhibitory effect on *Acta2* expression. **c, d** Real-time PCR analysis of *Ski* (**c**) and *Skil* (**d**) expression in *Tfeb*-silenced MECs or in doxycycline-treated MEC-TFEB S142A (dox) cells. **e** Real-time PCR analysis of *Skil* gene expression in MECs infected with a lentivirus expressing shRNA for *Skil*. **f** The inhibitory effect of TFEBS142A overexpression on *Acta2* transcription was not rescued by *Skil* silencing. *Acta2* expression was evaluated by real-time PCR in doxycycline-treated MEC-TFEBS142A (dox) cells transfected with lentivirus expressing shRNA for *Skil*. Values are shown as the mean ±SEM, n=2 (**c**, **f**), n=3 (**d**, *Tfeb* silencing experiment), n=6 (**d**, TFEB S142A overexpression experiment). Student's two-tailed t test p values are reported in the plots.

g Selection of shRNAs for *Tgif1* silencing under TFEB S142A overexpression conditions. Real-time PCR analysis of *Tgif1* mRNA in MEC-TFEBS142A cells infected with lentiviruses coding for three *Tgif1* shRNAs, where TFEB S142A overexpression was induced with doxycycline for 24 h (dox). Values are shown as the mean ±SEM, n=2. Student's two-tailed test p values are shown in the plot.

Source data are provided as a Source Data file.

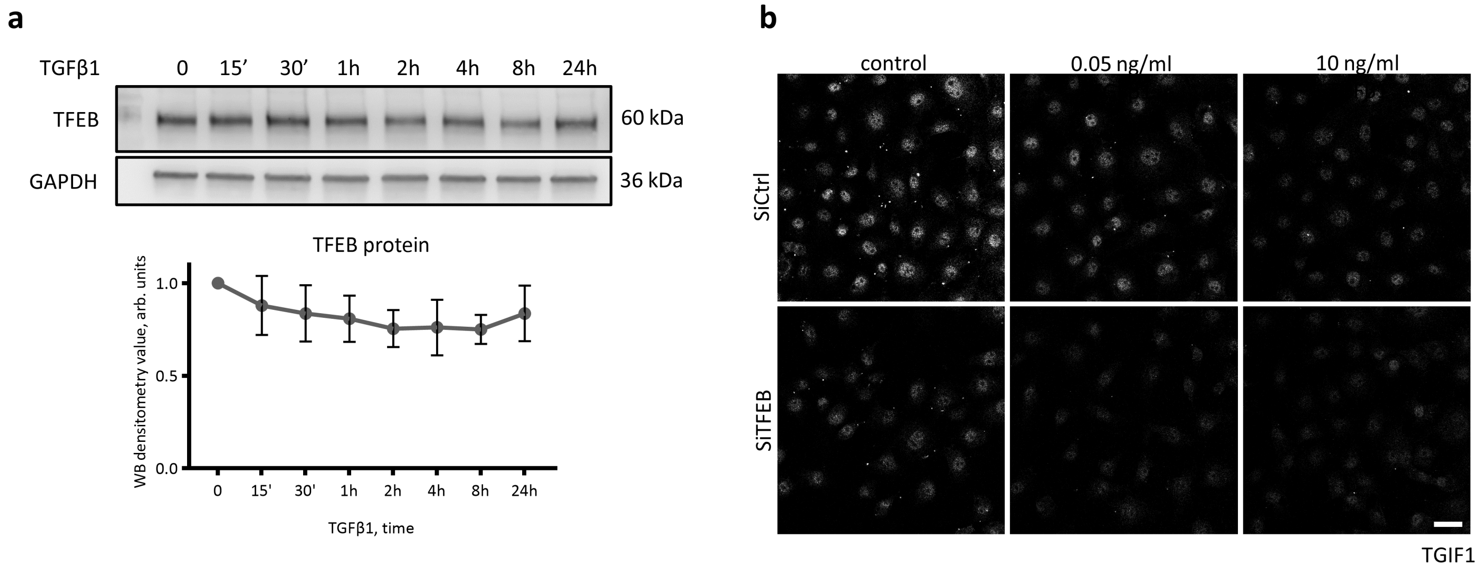

Supplementary Figure 9. Effects of TGFβ1 simulation on TFEB and TGIF1 protein levels

a TGFβ1 stimulation does not induce a significant TFEB protein quantity variation in MECs in the first 24 h.
Top, Western blot analysis of TFEB in MECs treated with TGFβ1 for the indicated time intervals. Bottom, densitometric analysis of Western blots. Values are shown as the mean ±SEM, n=3.

b Effect of *Tfeb* silencing on TGIF1 protein levels in MECs treated with low (0.05 ng/ml) and high (10 ng/ml) doses of TGFβ1 for 15 min. The scale bar is 25 µm. The experiment was repeated three times, the quantification is reported in the Fig. 8b.

Source data are provided as a Source Data file.

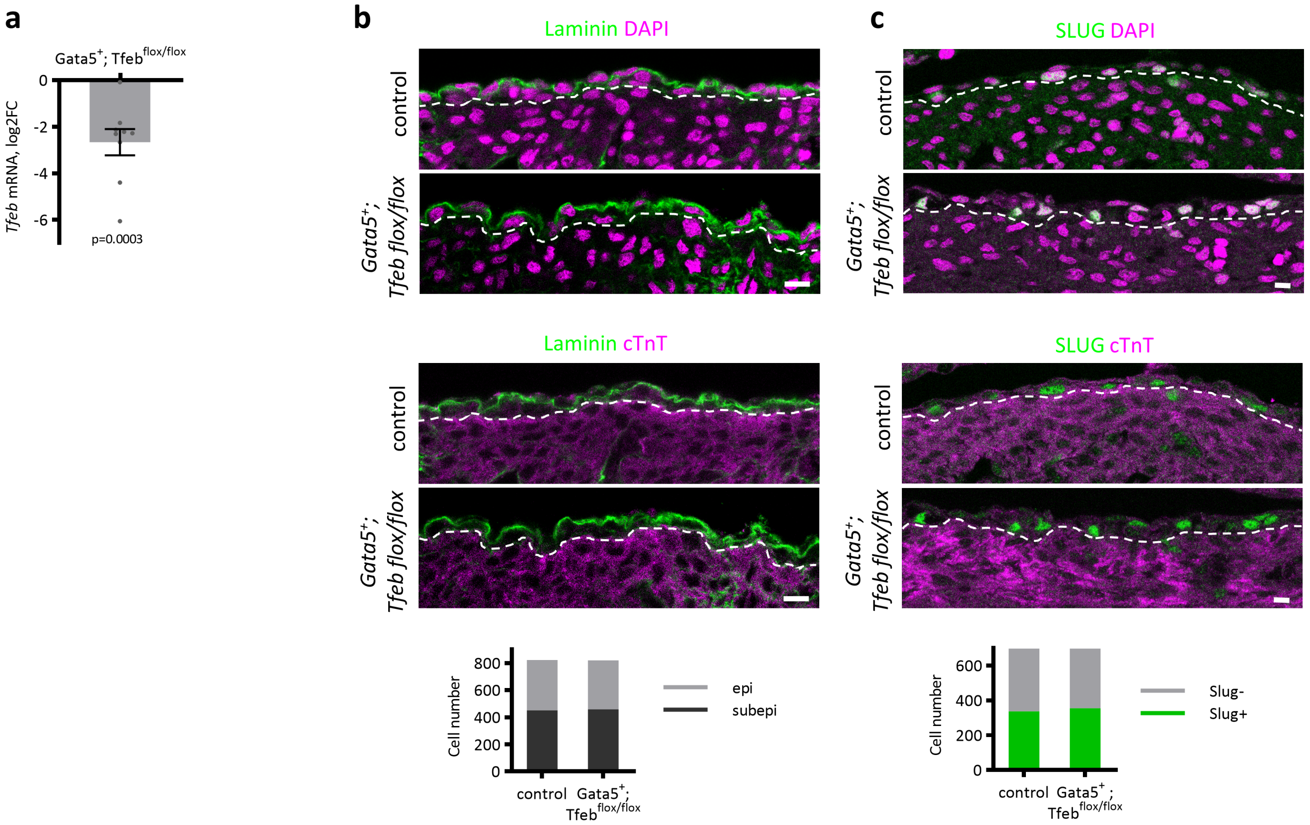

Supplementary Figure 10. *Tfeb* knockout in the epicardium did not influence EMT markers at E13.5

a Confirmation of *Tfeb* deletion in epicardial cells of *Gata5⁺; Tfeb* $^{flox/flox}$ embryos. Epicardial explants were cultured for 4 days in DMEM with 10% FCS, and *Tfeb* expression was analyzed by real-time PCR. Eight *Gata5⁺; Tfeb* $^{flox/flox}$ and 10 control explants were used for the experiment. Values are shown as the mean ±SEM, Student's t test p value is reported in the plot.

b-c Immunofluorescence analysis for laminin, Slug (green) and cardiac troponin C (magenta, bottom panels) in E13.5 Gata5+; Tfeb fs and control embryos. Nuclei are stained in magenta (top panels). The dashed line shows the myocardium surface. The scale bar is 10 µm. Five embryos of each genotype and at least 5 images for embryos were used for quantification in ImageJ. The total number of nuclei in all analyzed images is shown in the graphs. **b, bottom** Quantification of epithelial cells (localized above immunostained for laminin basal membrane) and delaminated subepicardial EPDCs (localized under basal membrane). **c, bottom** quantification of Slug-positive epicardial cells and EPDCs. Statistical significance was calculated with Fisher's exact test.

Source data are provided as a Source Data file.

Western blots membranes of Supplementary figures

Supplementary Figure 6a

The membrane was cut in two. The upper part was stained with anti-TFEB antibody, the lower part – with anti-GAPDH antibody.

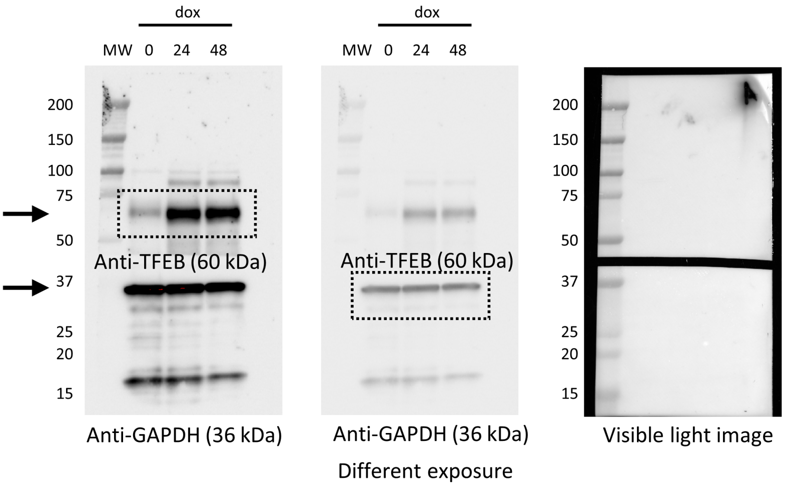

Supplementary Figure 6e

The membrane was first decorated with anti-TFEB antibody, then – with anti-GAPDH antibody.

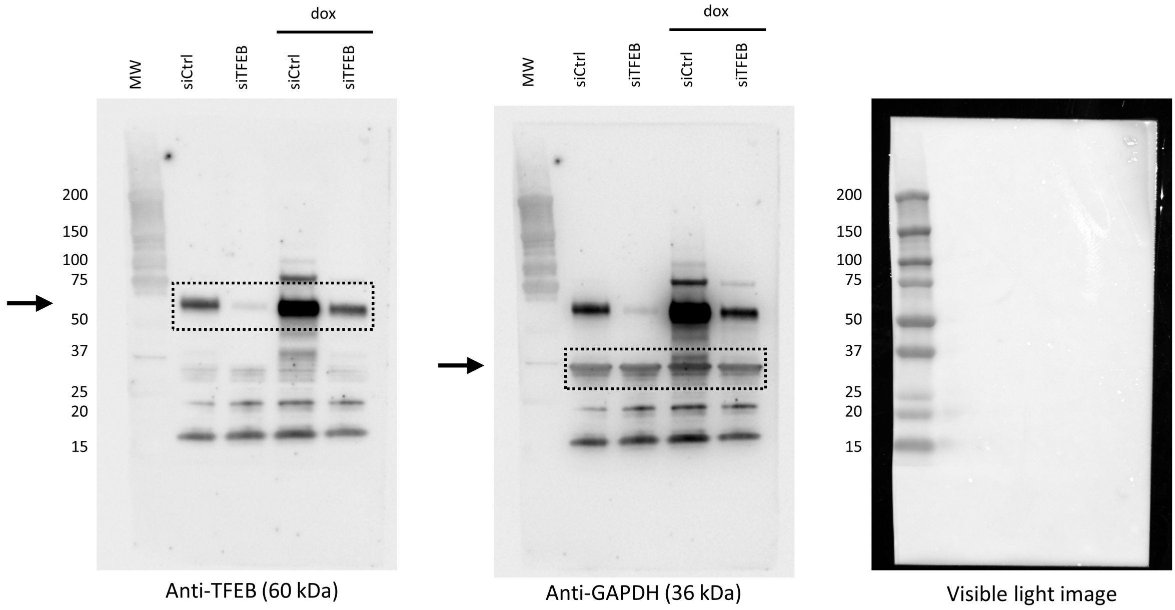

Supplementary Figure 9a

The membrane was first decorated with anti-TFEB antibody, then – with anti-GAPDH antibody.

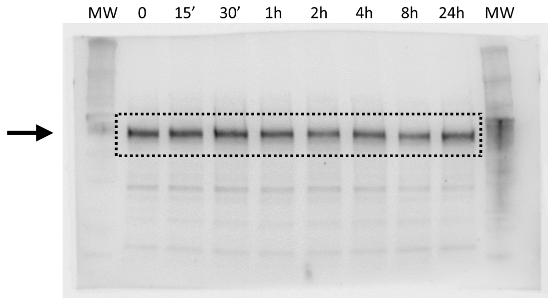

Anti-TFEB (60 kDa)

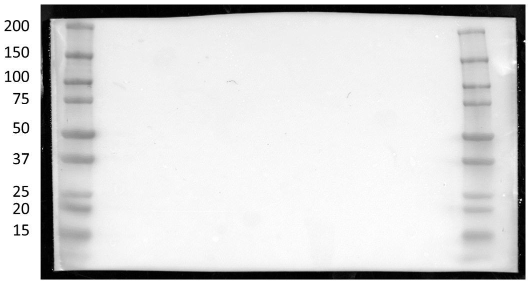

Visible light image

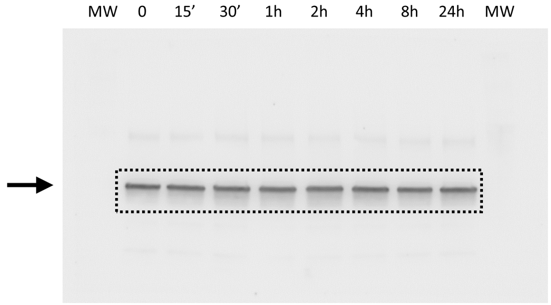

Anti-GAPDH (36 kDa)

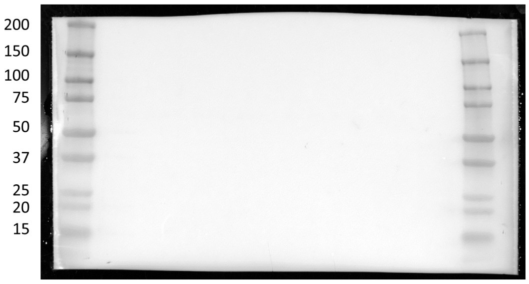

Visible light image

Supplementary Table 1

Antibody	Catalog number	Company	Application	Dilution	Validation
αSMA	A2547<br>1A4	Sigma-Aldrich	IF, WB<br>mouse, human,	IF 1:1000<br>WB 1:1000	Manufacturer's website
GAPDH	Ab8245<br>6C5	Abcam	WB<br>mouse, human	1:5000	Manufacturer's website
GFP	ab13970	Abcam	IF	1:1000	Manufacturer's website
GFP	A11122	Thermo Fisher Scientific	IHC	1:500	Manufacturer's website
CD31	550274	BD Pharmingen	IF<br>mouse	1:100	[1]
cTnT	MS-295-P	Thermo Fisher Scientific	IF mouse	1:100	Manufacturer's website
Cre Recombinase	15036<br>D7L7L	Cell Signaling Technology	IF	1:500	Manufacturer's website
Flag tag	14793<br>D6W5B	Cell Signaling Technology	IF	1:500	Manufacturer's website
PDGFRα	3174<br>D1E1E	Cell Signaling Technology	IF mouse	1:400	Manufacturer's website
PDGFRβ	28E1	Cell Signaling Technology	IF mouse	1:200	Manufacturer's website
Slug	C19G7	Cell Signaling Technology	IF mouse	1:100	Manufacturer's website
SM22α	ab14106	Abcam	IF human, mouse	1:200	Manufacturer's website
TBX18	ab115262	Abcam	IF mouse	1:100	[2]
TFEB	83010<br>E5P9M	Cell Signaling Technology	ChIP-seq mouse	5 µg for for $2x10^7$ cells	Manufacturer's website
TFEB	A303-673A	Bethyl Laboratories	WB, IF, human, mouse	WB 1:2000, IF 1:200	Manufacturer's website
TWIST	E7E2G, 31174	Cell Signaling Technology	IF mouse	1:400	Manufacturer's website
TGIF1	sc-9084<br>H-172	Santa Cruz Biotechnology	IF mouse	1:100	[3], Manufacturer's website
Vimentin	5741<br>D21H3	Cell Signaling Technology	IF mouse	1:200	Manufacturer's website
Vinculin	V9131	Sigma-Aldrich	WB human	1:2000	Manufacturer's website
WT1	sc-192<br>C19	Santa Cruz Biotechnology	IF mouse	1:100	[4], Manufacturer's website
ZEB1	E2G6Y<br>70512	Cell Signaling Technology	IF mouse		
ZO1	40-2200	Thermo Fisher Scientific	IF mouse	1:200	Manufacturer's website
HRP goat anti-mouse secondary Ab	115-035-003	Jackson ImmunoResearch Laboratories	WB	1:20000	
HRP goat anti-rabbit secondary Ab	111-035-003	Jackson ImmunoResearch Laboratories	WB	1:20000	
EnVision+ System- HRP Labelled Polymer Anti-Rabbit	K4003	Dako	IHC	Ready to use	
Alexa Fluor 555 donkey anti-mouse Ab	A31570	Thermo Fisher Scientific	IF	1:400	
Alexa Fluor 488 donkey anti-rabbit Ab	A21206	Thermo Fisher Scientific	IF	1:400	
Alexa Fluor 647 goat anti-rat	A21247	Thermo Fisher Scientific	IF	1:400	
Alexa Fluor 488 Goat anti-Chicken Ab	A-11039	Thermo Fisher Scientific	IF	1:400	

References

1. Zhang, L., et al. Single-cell transcriptomic profiling of lung endothelial cells identifies dynamic inflammatory and regenerative subpopulations. *JCI Insight* **7**(11) (2022).

2. Liu, B., et al. Inhibition of Notch Signaling Promotes the Differentiation of Epicardial Progenitor Cells into Adipocytes*. Stem Cells Int* 8859071 (2021).

3. Tateossian, H., et al. Otitis media in the Tgif knockout mouse implicates TGFβ signalling in chronic middle ear inflammatory disease*. Hum Mol Genet* **22**(13), 2553-2565 (2013).

4. Zhong, Y., et al. Arctigenin attenuates diabetic kidney disease through the activation of PP2A in podocytes*. Nat Commun* **10**(1), 4523 (2019).
