# Supplementary material for: The TFEB-TGIF1 axis regulates EMT in mouse epicardial cells
Source: Nat Commun. 2022 Sep 3;13:5191. doi: 10.1038/s41467-022-32855-3 (PMC9440911; doi:10.1038/s41467-022-32855-3)
Supplement: Supplementary file 2 — Reporting Summary [file 41467_2022_32855_MOESM2_ESM.pdf]

nature portfolio

Corresponding author(s): Elena Astanina

Last updated by author(s): Jul 29, 2022

Reporting Summary

Nature Portfolio wishes to improve the reproducibility of the work that we publish. This form provides structure for consistency and transparency in reporting. For further information on Nature Portfolio policies, see our Editorial Policies and the Editorial Policy Checklist.

Statistics

For all statistical analyses, confirm that the following items are present in the figure legend, table legend, main text, or Methods section.

n/a	Confirmed	
☐	☒	The exact sample size (*n*) for each experimental group/condition, given as a discrete number and unit of measurement
☐	☒	A statement on whether measurements were taken from distinct samples or whether the same sample was measured repeatedly
☐	☒	The statistical test(s) used AND whether they are one- or two-sided<br>*Only common tests should be described solely by name; describe more complex techniques in the Methods section.*
☒	☐	A description of all covariates tested
☐	☒	A description of any assumptions or corrections, such as tests of normality and adjustment for multiple comparisons
☐	☒	A full description of the statistical parameters including central tendency (e.g. means) or other basic estimates (e.g. regression coefficient) AND variation (e.g. standard deviation) or associated estimates of uncertainty (e.g. confidence intervals)
☐	☒	For null hypothesis testing, the test statistic (e.g. *F*, *t*, *r*) with confidence intervals, effect sizes, degrees of freedom and *P* value noted<br>*Give P values as exact values whenever suitable.*
☒	☐	For Bayesian analysis, information on the choice of priors and Markov chain Monte Carlo settings
☒	☐	For hierarchical and complex designs, identification of the appropriate level for tests and full reporting of outcomes
☒	☐	Estimates of effect sizes (e.g. Cohen's *d*, Pearson's *r*), indicating how they were calculated

Our web collection on statistics for biologists contains articles on many of the points above.

Software and code

Policy information about availability of computer code

Data collection	Leica TCS SPE or TCS SP8, LAS AF 2.6.3<br>7500 Real-time PCR System (Thermo Fisher Scientific)
Data analysis	ImageJ 1.51p<br>Image Lab 6.0.1 (Bio-Rad)<br>MACS 1.4.1<br>HOMER 4.10.3<br>GREAT 3.0.0<br>Data Viewer 1.5.4.6.<br>NRecon 1.4.4<br>CTVox 3.3.0<br>GraphPad Prism 9.3.1

For manuscripts utilizing custom algorithms or software that are central to the research but not yet described in published literature, software must be made available to editors and reviewers. We strongly encourage code deposition in a community repository (e.g. GitHub). See the Nature Portfolio guidelines for submitting code & software for further information.

Data

Policy information about availability of data

All manuscripts must include a data availability statement. This statement should provide the following information, where applicable:

- Accession codes, unique identifiers, or web links for publicly available datasets
- A description of any restrictions on data availability
- For clinical datasets or third party data, please ensure that the statement adheres to our policy

The ChIPseq dataset is available in The Gene Expression Omnibus of the National Center for Biotechnology Information (accession number GSE178575, [https://www.ncbi.nlm.nih.gov/geo/query/acc.cgi?acc=GSE178575]). The databases used in this study are available online: Jaspar [https://jaspar.genereg.net/], GREAT [http://great.stanford.edu/public/html/], HOMER [http://homer.ucsd.edu/homer/]. Source data for graphs and plots are provided as Source data file with this paper.

Human research participants

Policy information about studies involving human research participants and Sex and Gender in Research.

Reporting on sex and gender	We were not informed about the sex of the babies from whom umbilical cords were obtained. We generated HUVECs from a pool of donors of mixed sex.
Population characteristics	The primary vein ECs from umbilical cords were obtained from informed healthy pregnant women.
Recruitment	Informed consent was obtained from each patient.
Ethics oversight	The isolation of primary human ECs was approved by the Office of the General Director and Ethics Committee of the Azienda Sanitaria Ospedaliera Ordine Mauriziano of Torino Hospital (protocol approval no. 586, October 22, 2012; no. 26884, August 28, 2014; and no. 1494 del July 9 2018).

Note that full information on the approval of the study protocol must also be provided in the manuscript.

Field-specific reporting

Please select the one below that is the best fit for your research. If you are not sure, read the appropriate sections before making your selection.

☒ Life sciences ☐ Behavioural & social sciences ☐ Ecological, evolutionary & environmental sciences

For a reference copy of the document with all sections, see nature.com/documents/nr-reporting-summary-flat.pdf

Life sciences study design

All studies must disclose on these points even when the disclosure is negative.

Sample size

In the articles where epicardium biology was investigated with methods similar to ours and which we cyte in our manuscript ([https://doi.org/10.1016/j.ydbio.2008.07.038], [https://doi.org/10.1016/j.ydbio.2011.05.668], [https://doi.org/10.1242/dev.093385], [https://doi.org/10.1242/dev.060996], [https://doi.org/10.1152/ajplung.00212.2018]) sample size was 3-6 embryos per group.
In the analysis of mouse embryos, we aimed to reduce the number of pregnant mice to sacrifice for the ethical reasons.
Tfeb expression analysis in TfebEGFP embryos (Fig. 1b-d). We performed pilot experiments and observed a strong TFEB-EGFP expression and high similarity between transgenic embryos. In our analysis we counted cells (positive and negative for EMT TFs or epithelial and delaminated), therefore from one embryo we already obtained a high number of cells for quantification. As we observed practically no variability between the data from different transgenic embryos, we stopped collecting embryos after we collected 1 litter of transgenic embryos and 1 litter of wild type embryos for a time point (therefore 8 total pregnant females were used).
Gata5+; Tfeb fs embryos constituted 25% of all embryos because the parents were kept as heterozygotes.
For micro-CT analysis of the morphological defects of Gata5+; Tfeb fs embryos we used groups of 8 embryos because we had no hypothesis what kind of difference to expect.
Gata5+; Tfeb fs embryos phenotype analysis by IF staining on embryo tissue (Fig. 3e-f). We performed pilot experiments and observed a striking defect in mutant embryos (reduced number of fibroblasts and SMCs in myocardium), visible to the naked eye, and a very low variability between transgenic embryos. We concluded that Gata5-Cre expression had high penetrance. This was also confirmed by the mortality data, all mutant embryos died at the same day of development. We supposed the sample size of 5 embryos for a group would be sufficient to determine the difference between groups.
The same rationale was applied to the phenotype characterization of Gata5+; Tfeb flox/flox embryos. They too constitute 25% of embryos in a litter. We supposed that, using the same Gata5-Cre driver, we would obtain the same low variability between embryos. In fact, we found a highly significant difference in numbers of PDGFRα+ and PDGFRβ+ cells migrated in the myocardium (Fig. 8c).
For the experiments with epicardial explants (Fig. 1e, 4b, 6e, 8e), we had to use a reduced number of explants (4-6, in a few cases 3) per sample, because of the logistical and ethical reasons. To perform an experiment, we needed all the explants (transgenic and control) ready at the same time, to culture, treat, fix and immunostain them simultaneously to reduce the variability. For an experiment consisted of 2 experimental points (ctrl and TGFβ-treated), if consider 4 explants per group, 8 transgenic embryonic hearts were needed. Taken into

consideration their incidence and number of pups in a litter, we needed 6 females got pregnant on the same day. It would have been unfeasible to increase the number of the explants. To efficiently use all the material available, we carefully took images of all the cells in an explant and used all these images for quantification.
Other in vitro experiments were performed in cell lines, therefore biological replicates showed high reproducibility. Taken into consideration this fact and our experience in the assays (gene expression analysis, Western blots, IF quantification) we concluded that triplicates would suffice to produce statistically significant data.

In the articles where epicardium biology was investigated with methods similar to ours and which we cyte in our manuscript ([https://doi.org/10.1016/j.ydbio.2008.07.038], [https://doi.org/10.1016/j.ydbio.2011.05.668], [https://doi.org/10.1242/dev.093385], [https://doi.org/10.1242/dev.060996], [https://doi.org/10.1152/ajplung.00212.2018]) sample size was 3-6 embryos per group.
In the analysis of mouse embryos, we aimed to reduce the number of pregnant mice to sacrifice for the ethical reasons.
Tfeb expression analysis in TfebEGFP embryos (Fig. 1b-d). We performed pilot experiments and observed a strong TFEB-EGFP expression and high similarity between transgenic embryos. In our analysis we counted cells (positive and negative for EMT TFs or epithelial and delaminated), therefore from one embryo we already obtained a high number of cells for quantification. As we observed practically no variability between the data from different transgenic embryos, we stopped collecting embryos after we collected 1 litter of transgenic embryos and 1 litter of wild type embryos for a time point (therefore 8 total pregnant females were used).
Gata5+; Tfeb fs embryos constituted 25% of all embryos because the parents were kept as heterozygotes.
For micro-CT analysis of the morphological defects of Gata5+; Tfeb fs embryos we used groups of 8 embryos because we had no hypothesis what kind of difference to expect.
Gata5+; Tfeb fs embryos phenotype analysis by IF staining on embryo tissue (Fig. 3e-f). We performed pilot experiments and observed a striking defect in mutant embryos (reduced number of fibroblasts and SMCs in myocardium), visible to the naked eye, and a very low variability between transgenic embryos. We concluded that Gata5-Cre expression had high penetrance. This was also confirmed by the mortality data, all mutant embryos died at the same day of development. We supposed the sample size of 5 embryos for a group would be sufficient to determine the difference between groups.
The same rationale was applied to the phenotype characterization of Gata5+; Tfeb flox/flox embryos. They too constitute 25% of embryos in a litter. We supposed that, using the same Gata5-Cre driver, we would obtain the same low variability between embryos. In fact, we found a highly significant difference in numbers of PDGFRα+ and PDGFRβ+ cells migrated in the myocardium (Fig. 8c).
For the experiments with epicardial explants (Fig. 1e, 4b, 6e, 8e), we had to use a reduced number of explants (4-6, in a few cases 3) per sample, because of the logistical and ethical reasons. To perform an experiment, we needed all the explants (transgenic and control) ready at the same time, to culture, treat, fix and immunostain them simultaneously to reduce the variability. For an experiment consisted of 2 experimental points (ctrl and TGFβ-treated), if consider 4 explants per group, 8 transgenic embryonic hearts were needed. Taken into consideration their incidence and number of pups in a litter, we needed 6 females got pregnant on the same day. It would have been unfeasible to increase the number of the explants. To efficiently use all the material available, we carefully took images of all the cells in an explant and used all these images for quantification.
Other in vitro experiments were performed in cell lines, therefore biological replicates showed high reproducibility. Taken into consideration this fact and our experience in the assays (gene expression analysis, Western blots, IF quantification) we concluded that triplicates would suffice to produce statistically significant data.

Data exclusions

No data were excluded from analysis except for obvious technical failures of experiments

Replication

For in vivo experiments, different embryos were considered biological replicates. For the experiments including primary epicardial cells, a pool of cells grown from one embryo heart was considered a biological replicate. Number of embryos used in each analysis is indicated in figure legends. For in vitro experiments, experiments were replicated at least three times.

Randomization

We did not randomize the samples because our experimental design did not require this type of strategy.

Blinding

The investigators were not blinded to the allocation of the samples during the experiments and analyses because full blinding would be unfeasible in the laboratory setting when often the same researcher collects, proceeds and analyzes data. We aimed to avoid bias by applicating the same criteria and procedure (e.g. threshod application for microscopy images analysis) for different groups of samples. In other experiments the objective or numerical nature of the data (Western blots, real-time PCR) prevents the influence of the researcher.

Reporting for specific materials, systems and methods

We require information from authors about some types of materials, experimental systems and methods used in many studies. Here, indicate whether each material, system or method listed is relevant to your study. If you are not sure if a list item applies to your research, read the appropriate section before selecting a response.

Materials & experimental systems

n/a	Involved in the study	
☐	☒	Antibodies
☐	☒	Eukaryotic cell lines
☒	☐	Palaeontology and archaeology
☐	☒	Animals and other organisms
☒	☐	Clinical data
☒	☐	Dual use research of concern

Methods

n/a	Involved in the study	
☐	☒	ChIP-seq
☒	☐	Flow cytometry
☒	☐	MRI-based neuroimaging

Antibodies

Antibodies used	Anti-GAPDH (6C5, ab8245, mouse, human, WB 1:1000, IF 1:1000), anti-GFP (ab13970, IF 1:1000), anti-SM22α (ab14106, mouse, human, IF 1:200, WB 1:2000), and anti-TBX18 (ab115262, mouse, IF 1:100, https://doi.org/10.1155/2021/8859071) were purchased from Abcam. Anti-CD31 (550274, mouse, IF 1:100) was purchased from BD Pharmingen. Anti-TFEB (A303-673A, mouse, human, WB 1:2000, IF 1:200) was purchased from Bethyl Laboratories. Anti-Cre Recombinase (D7L7L, 15036, IF 1:500), anti-Flag tag (D6W5B, 14793, IF 1:500), anti-PDGFRα (D1E1E, 3174, mouse, IF 1:400), anti-PDGFRβ (28E1, 3169, mouse, IF 1:200), anti-Slug (C19G7, 9585, mouse, IF, 1:100), anti-TFEB (E5P9M, 83010, mouse, ChIPseq, 5 μg for for 2x10^7 cells), anti-TWIST1 (E7E2G, 31174, mouse, IF 1:400), anti-Vimentin (D21H3, 5741, mouse, IF 1:200), anti-ZEB1 (E2G6Y, 70512, mouse, IF 1:400) were purchased from Cell Signaling Technology. Anti-TGIF1 (H-172, sc-9084, mouse, IF 1:100, https://doi.org/10.1093/hmg/ddt103) and anti-WT1 (C19, sc-192, mouse, IF 1:100) were purchased from Santa Cruz Biotechnology. Anti-αSMA (1A4, A2547, mouse, human, WB 1:1000, IF 1:1000) and anti-vinculin (V9131, human, WB 1:1000) were purchased from Sigma-Aldrich. Anti-GFP (A11122, IHC 1:500), anti-cTnT (MS-295, mouse, IF 1:100), and anti-ZO1 (40-2200, mouse, IF 1:200) were purchased from Thermo Fisher Scientific. HRP goat anti-mouse and goat anti-rabbit secondary antibodies (WB 1:20000) were purchased from Jackson ImmunoResearch Laboratories. EnVision+ System- HRP Labelled Polymer Anti-Rabbit (IHC ready-to-use) was purchased from Dako. Alexa Fluor 555 donkey anti-mouse, Alexa Fluor 488 donkey anti-rabbit, Alexa Fluor 647 goat anti-rat and Alexa Fluor 488 Goat anti-Chicken secondary antibodies (IF 1:400) were purchased from Thermo Fisher Scientific.
Validation	We chose well-referenced antibodies. For each combination of species (mouse or human) and application (WB, IF, IHC, ChIPseq) we checked if the manufacturer approves the usage. For the antibodies no longer in production we report the relevant citations. We validated that the result (the molecular weight of the band in Western blot or the staining localization in IF and IHC) is in line with the expected result based on literature data, our experience and images provided by the manufacturer.

Eukaryotic cell lines

Policy information about cell lines and Sex and Gender in Research

Cell line source(s)	The Madin Darby canine kidney cell line (MDCK) was purchased from ATCC (CCL-34). Mouse embryonic epicardial cell line (MEC) was derived from a pool of 6 embryonic hearts, not distinguished for sex, so it has a mixed sex origin. The protocol to prepare and use human ECs was approved by the Office of the General Director and Ethics Committee of the Azienda Sanitaria Ospedaliera Ordine Mauriziano of Torino Hospital (protocol approval no. 586, Oct 22 2012 and no. 26884, Aug 28 2014 and no. 1494, July 9 2018) and informed consent was obtained from each donor.
Authentication	Cells were authenticated by validation the morphology at microscope.
Mycoplasma contamination	All cell cultures were routinely tested for micoplasma contamination and resulted negative.
Commonly misidentified lines (See ICLAC register)	No commonly misidentified cell lines were used in this study.

Animals and other research organisms

Policy information about studies involving animals; ARRIVE guidelines recommended for reporting animal research, and Sex and Gender in Research

Laboratory animals	Tfeb-flagfs mice, Tfeb flox/flox mice, TfebEGFP mice and Gata5-Cre mice on C57BL/6 background were used in this study. References for each mouse line are included in the manuscript (Methods section). To generate embryos, females and males of appropriate genotypes aged between 9 and 18 weeks were mated. 8 weeks old wild-type and TfebEGFP mice were used for analysis in the adults.
Wild animals	The study did not involve wild animals.
Reporting on sex	The phenotypes of mice described in the manuscript did not depend on sex, therefore, embryos of both sexes were included in the study.
Field-collected samples	The study did not involve samples collected in the field.
Ethics oversight	All animal procedures were approved by the ethics committee of the University of Turin and by the Italian Ministry of Health (protocol approval no. 864/2015-PR).

Note that full information on the approval of the study protocol must also be provided in the manuscript.

ChIP-seq

Data deposition

☒ Confirm that both raw and final processed data have been deposited in a public database such as GEO.

☒ Confirm that you have deposited or provided access to graph files (e.g. BED files) for the called peaks.

Data access links *May remain private before publication.*	The ChIPseq dataset is available in The Gene Expression Omnibus of the National Center for Biotechnology Information (accession number GSE178575, direct link: https://www.ncbi.nlm.nih.gov/geo/submission/update/?acc=GSE178575).

Files in database submission

Four data files where provided with the submission to GEO:

- TFEB_mouse.Sample_ChIP_IgG_R1_e5_nolambda_peaks.bed (MACS peaks on IgG sample, total = 1343 peaks)

- TFEB_mouse.Sample_ChIP_TFEB_R1_e5_nolambda_peaks.bed (MACS peaks on TFEB sample, total = 8258 peaks)

- TFEB_mouse.Sample_ChIP_IgG_R1.fastq.gz (raw reads on IgG sample, total = 10077770 reads, single-end with read length=50 nt)

- TFEB_mouse.Sample_ChIP_TFEB_R1.fastq.gz (raw reads in TFEB sample, total = 15903002 reads, single-end with read length=50 nt)

Genome browser session
(e.g. UCSC)

Not applicable

Methodology

Replicates

No replicates were run at genome wide level.

Sequencing depth

Here is the information about number of reads and read length.

- TFEB_mouse.Sample_ChIP_IgG_R1.fastq.gz (raw reads on IgG sample, total = 10077770 reads, single-end with read length=50 nt)

- TFEB_mouse.Sample_ChIP_TFEB_R1.fastq.gz (raw reads in TFEB sample, total = 15903002 reads, single-end with read length=50 nt)

Antibodies

TFEB (D2O7D) Rabbit mAb #37785
Rabbit IgG, polyclonal - Isotype Control (ab37415)

Peak calling parameters

macs14 -t TFEB.sam --format=SAM --nolambda --gsize=mm -n TFEB --pvalue=1e-5

Data quality

Peaks quality metrics is generated by using MACS software

Software

ChIP-seq reads were aligned to the mm9 genome assembly using Bowtie v0.12.7 with the following parameters: -q –max/dev/null -v 1 -S –sam-nohead -m 1. Duplicate reads were filtered out. BedGraph files were generated by using the MACS tool. Peak calling was performed by MACS14 using a p value cutoff = 1E-05. HOMER and GREAT software were used for ChIP-seq peak annotation and analysis, while the Jaspar database was used as a source for the localization of TFEB binding sites
