# Supplementary material for: The TFEB-TGIF1 axis regulates EMT in mouse epicardial cells
Source: Nat Commun. 2022 Sep 3;13:5191. doi: 10.1038/s41467-022-32855-3 (PMC9440911; doi:10.1038/s41467-022-32855-3)
Supplement: Supplementary file 3 — Source Data [file 41467_2022_32855_MOESM3_ESM.zip › Source data/Western blots.pdf]

Western blots membranes

Figure 2c

The samples (the same volume) were loaded in the same gel twice, then the membrane was cut in two parts and developed in parallel (left – stained with anti-TFEB antibody, right – stained with anti-GAPDH antibody).

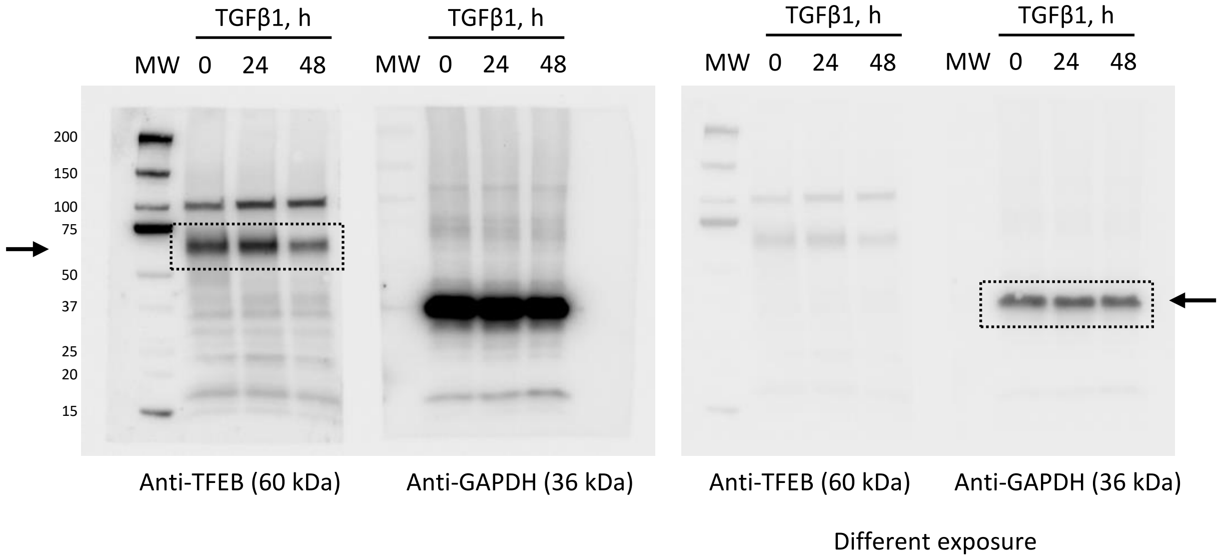

Figure 4e

First membrane was decorated first with anti-αSMA antibody, then – with anti-GAPDH antibody.

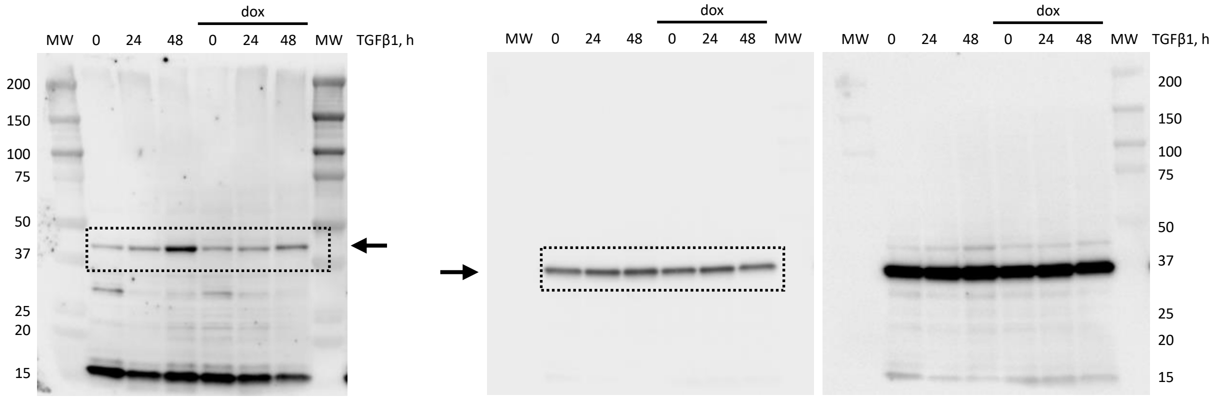

Anti-αSMA (45 kDa)

Anti-GAPDH (36 kDa)

Anti-GAPDH (36 kDa), different exposure

Second membrane (the same volume of the same samples was loaded in a second gel) was decorated with anti-SM22α antibody, then – with anti-GAPDH antibody. This image of GAPDH was not included in the manuscript figure, because it is the same that GAPDH staining on the αSMA blot shown above.

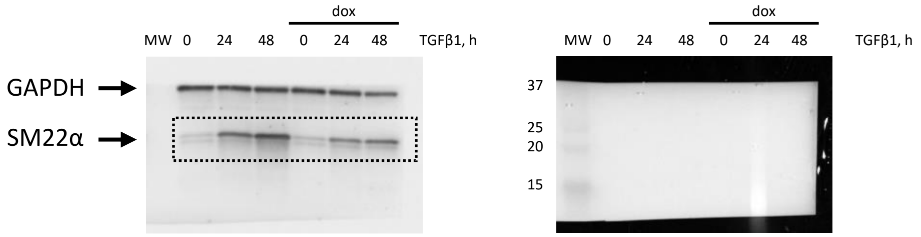

Anti-GAPDH (36 kDa)
Anti-SM22α (23 kDa)

Visible light image

Figure 4g

The membrane was cut in two. The upper part was stained with anti-TFEB antibody, the lower part – first, with anti-GAPDH antibody, then – with anti-SM22α antibody.

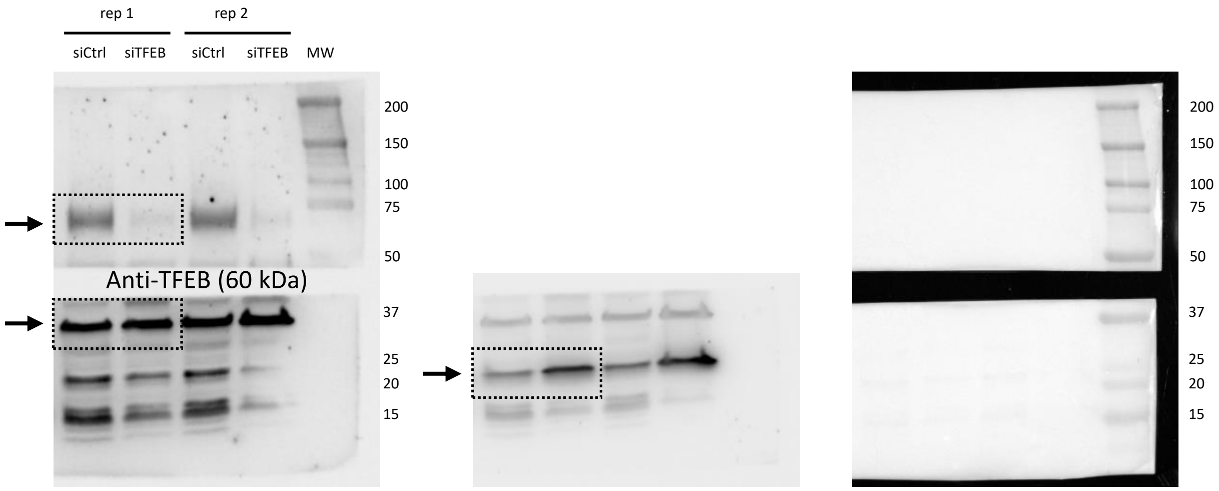

Anti-GAPDH (36 kDa) Anti-SM22α (23 kDa) Visible light image

The same volume of the same samples was loaded into second gel, which was stained with anti-αSMA antibody.

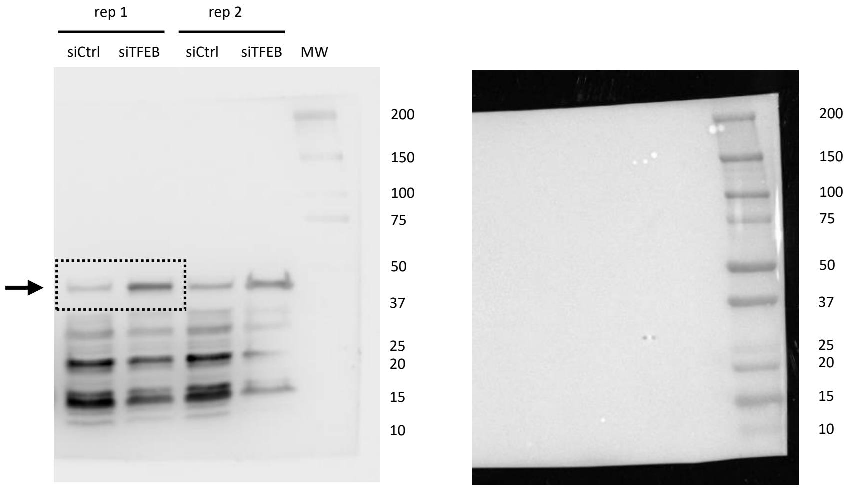

Anti-αSMA (45 kDa) Visible light image

Figure 9a

First membrane was decorated first with anti-TFEB antibody.

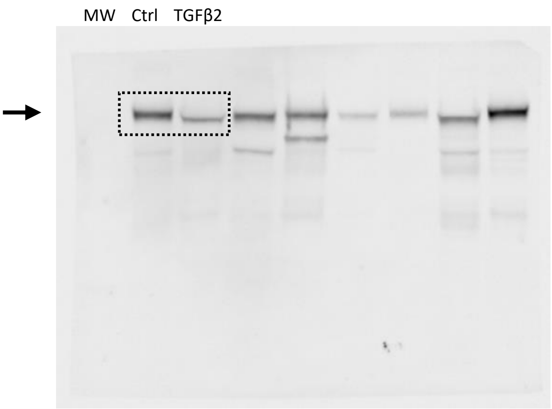

Anti-TFEB (60 kDa)

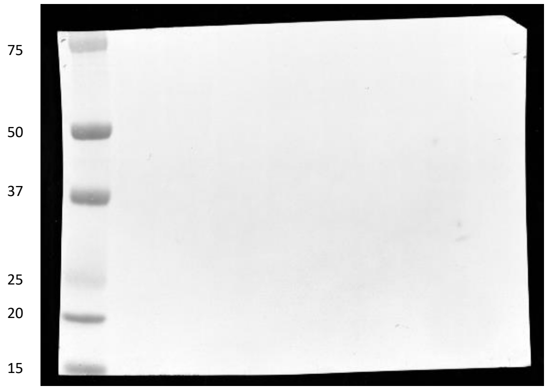

Visible light image

Second membrane (the same volume of the same samples was loaded in a second gel and processed in parallel) was decorated with anti-vinculin antibody

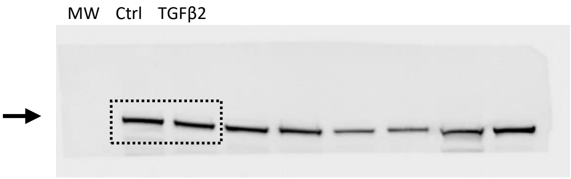

Anti-Vinculin (120 kDa)

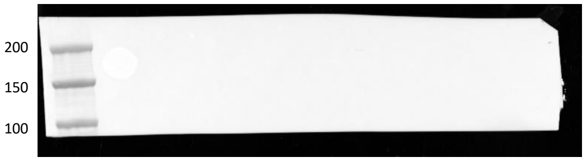

Visible light image

Figure 9b

The membrane was cut in two. The upper part was stained with anti-TFEB antibody, the lower part – with anti-SM22αantibody. The 2d and 7° lanes (cells cultured in complete medium) were not reported in the manuscript figure.

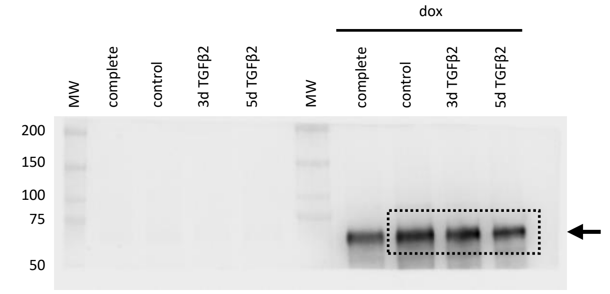

Anti-TFEB (60 kDa)

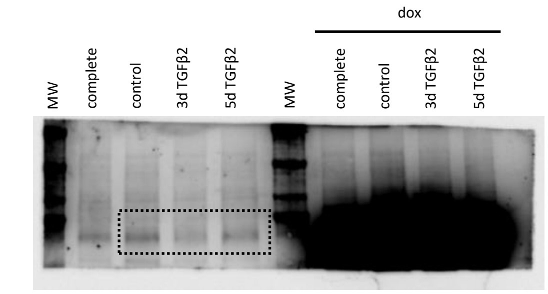

Anti-TFEB (60 kDa), different exposure

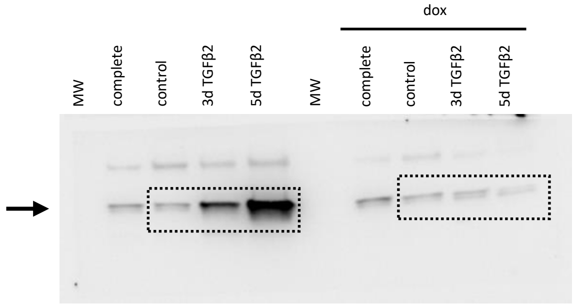

Anti-SM22α (23 kDa)

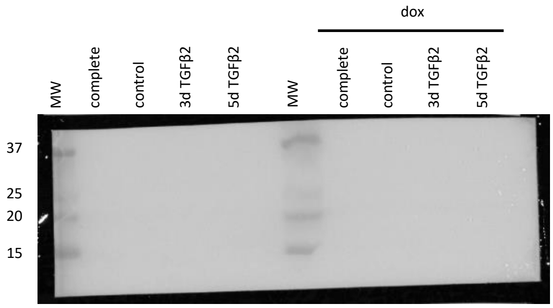

Visible light image

The same volume of the same samples was loaded into second gel, which was stained first with anti-αSMA antibody and after – with anti-GAPDH antibody.

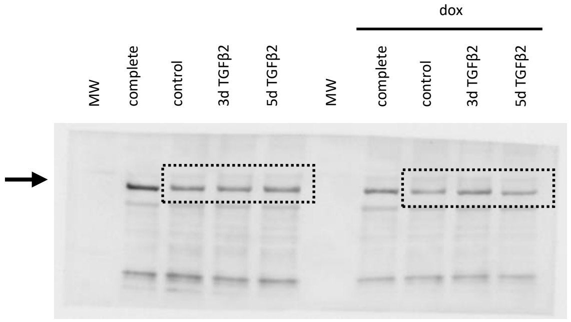

Anti-αSMA (45 kDa)

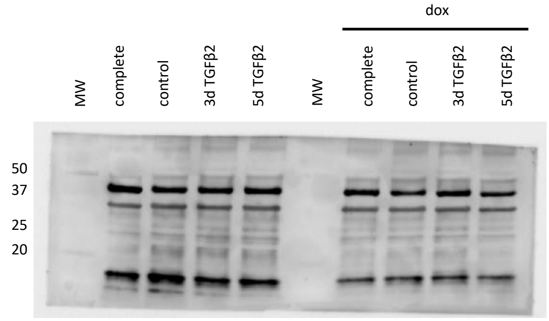

Anti-αSMA (45 kDa), different exposure

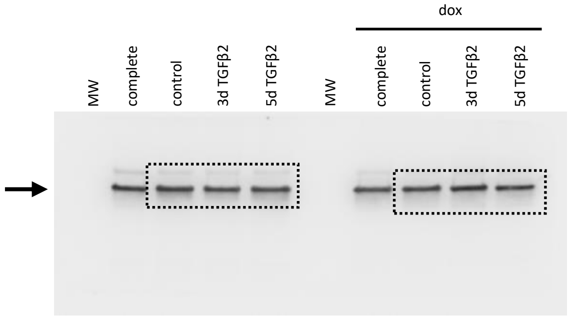

Anti-GAPDH (36 kDa)

Figure 9c

First membrane was decorated first with anti-αSMA antibody, then – with anti-GAPDH antibody. The 2d and 6th lanes (cells cultured in complete medium) were not reported in the manuscript figure.

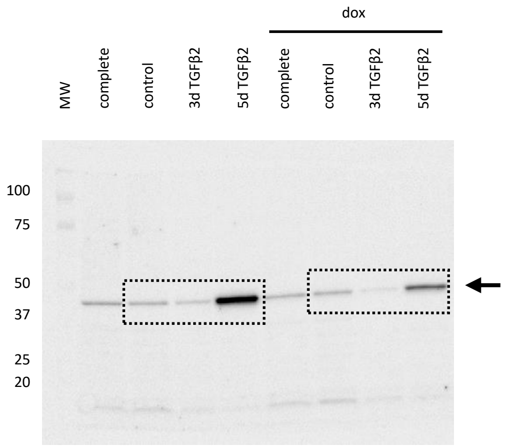

Anti-αSMA (45 kDa)

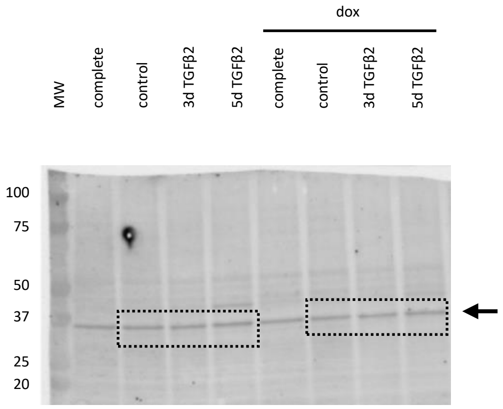

Anti-GAPDH (36 kDa)

The same volume of the same samples was loaded into second gel, which was then stained with anti-TFEB antibody.

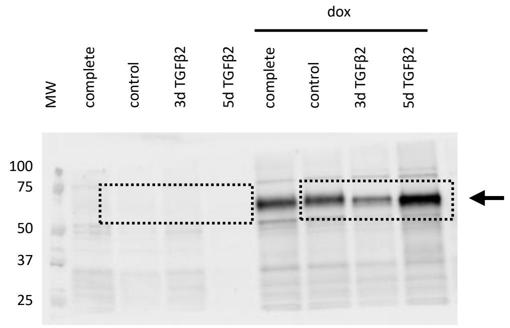

Anti-TFEB (60 kDa)

The same volume of the same samples was loaded into the third gel, which was then stained with anti-SM22α antibody.

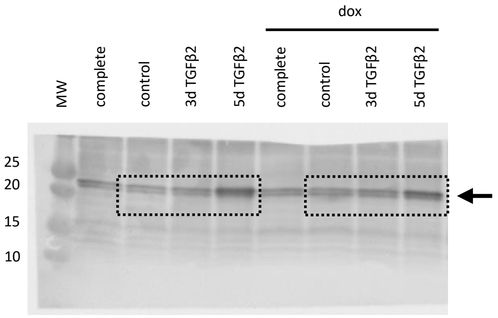

Anti-SM22α (23 kDa)
